# Far-red LED light alters circadian rhythms and elicits dark-adapted ERG responses in rodents

**DOI:** 10.1371/journal.pone.0326710

**Published:** 2025-07-01

**Authors:** Xian Chen, Steven Kreuser, Dinesh Hirenallur-Shanthappa

**Affiliations:** 1 Global-Discovery, Investigative and Translational Sciences, Cambridge, Massachusetts, United States of America; 2 Comparative Medicine, Pfizer Research and Development, Groton, Connecticut, United States of America; University of Texas Southwestern Medical Center, UNITED STATES OF AMERICA

## Abstract

Rodents are assumed to be blind to red light, thus red light is often used in the dark phase of a light/dark cycle to facilitate study procedures using nocturnal rodents. However, effects of red light in dark phase on behaviors and circadian rhythms in rodents are not yet clear. Thus, we evaluated effects of various long wavelength red light-emitting diode (LED) light on circadian rhythm and electroretinogram (ERG) in C57BL/6J mice and Wistar Han rats. Animals were implanted with telemetry devices to measure body temperature, heart rate, blood pressure, and locomotor activity for circadian rhythm assessment. In contrary to infra-red light, all visible long wavelength red lights, including the far-red LED light with a peak at 741 nm, induced significant alterations in circadian rhythms and dark-adapted rod photoreceptor-mediated ERG responses in mice and/or rats. However, far-red light did not elicit light-adapted cone photoreceptor-mediated ERG responses in both mice and rats. These findings demonstrate that rodents can perceive all spectrum of long wavelength red lights that are visible to humans, and exposures of red lights in dark phase interfere with their circadian rhythms. A dim far-red LED with peak wavelength in the range of 740–760 nm is recommended to use in the dark phase of a rodent room, and potential impacts are considered when using red light >2 photopic lux.

## Introduction

Light is the most important zeitgeber of circadian rhythms in humans and animals, influencing their sleep, heart rate, blood pressure, body temperature, locomotor activity, endocrine, and even metabolism [1–3]. Light is essential for image-forming (IF) vision, which is crucial for sighted animals to detect and distinguish surrounding objects, as well as for non-image-forming (NIF) processes such as the entrainment of circadian rhythm, the alertness in diurnal animals and humans, and the acute induction of sleep in nocturnal animals [4–6]. Thus, light spectrum, intensity, and the timing of light exposures are also critical for the well-being of different mammalian species. Exposure to light at night (LAN) can significantly alter animals circadian rhythm and compromise animal welfare and reproducibility of research results [7–11]. In humans, exposure to light at night has been shown to suppress melatonin synthesis and interrupt circadian rhythms, implicating sleep, performance, mental health, and safety [12–14].

Mice and rats are commonly used animal models in biomedical research. These animals are nocturnal, which is in contrary to diurnal humans, and are very sensitive to low intensity light exposures at night [2,15]. Thus, testing animals during their inactive period (light phase) may produce incorrect results due to cognitive deficits, lack of motivation to perform the task, and stress from being disturbed during their sleep period [16,17]. Therefore husbandry and specific experimental procedures are often conducted during animals’ active period (dark phase) with red light to facilitate procedures under a reverse light cycle to minimize stress and optimize data quality and translatability [18].

Mice and rats have rod photoreceptor-dominant retina, with cone photoreceptor making up as little as 3% and 1% of their photoreceptors, respectively [19,20]. The rod opsin (rhodopsin) exhibits a peak sensitivity at 498 nm [21]. These animals have a dichromatic vision, which is in contrary to the trichromatic vision in humans [22]. The two sets of cones in mice and rats have peak sensitivity to the ultraviolet (UV) spectrum (UV cones or UV sensitive cones with peak absorption at ~360 nm) and the green spectrum (M cones or middle wavelength sensitive cones with peak absorption at ~ 510 nm) [19,23–25]. Due to the lack of long wavelength sensitive cones (red cones with peak absorption at ~ 560 nm) [15,26], rodents are generally considered to be less sensitive to longer wavelength red light than humans [15]. Recently, light-adapted spectral sensitivity curves of retinal photoreceptors were determined in mice and rats by a constant response electroretinogram (ERG), where response amplitudes were kept constant by adjusting light intensities throughout the UV-visible spectrum (i.e., 300–700 nm) [27]. These measurements identified two peaks in mice at 359 nm and 511 nm, and in rats at 362 nm and 502 nm, corresponding to ultraviolet and M cones, respectively. No significant light-adapted ERG responses were detected for long wavelength light flashes above 620 nm. In addition to rods and cones, light is also perceived by the non-image-forming intrinsically photosensitive retinal ganglion cells (ipRGCs) located in the inner retina. These ipRGCs contains photopigment melanopsin, which is sensitive to blue light with peak absorption at 480 nm and insensitive to red light above 620 nm [28,29].

However, there is increasing evidence from different laboratories challenging the assumption that the red light is perceived by rodents as darkness. Exposure to high intensity (1040 µW/cm^2^) red light (600 nm or higher) during middle of the night was as effective as white light (780 µW/cm^2^) in suppressing N-acetyltransferase activity and pineal and circulating melatonin in rats [30,31]. Even dim (1 lux) red LED light (peak at 652 nm) triggered by activity was sufficient to increase the circadian period in mice compared to constant darkness [32] and red light at intensities above 10 lux altered their sleep-wake behavior [33]. Moreover, long-term exposure of low intensity red light at night (> 620 nm at 8 lux) induced significant alterations in circadian metabolism and physiology in rats, including decreased plasma melatonin levels and significantly disrupted circadian rhythms of total fatty acid, lactic acid, glucose, insulin, leptin, corticosterone, pO_2_, and pCO_2_ in the plasma compared with those obtained in the control condition [34]. Recently, rats were shown to have significant dark-adapted and light-adapted ERG responses to 656 nm (*λ*_p_) LED light at low intensities [22] and they were capable of discriminating objects even under 729 nm (*λ*_p_) far-red LED light [35]. The light spectrum from the 741 nm (*λ*_p_) far-red LED is at the margin that human can perceive with naked eyes in the darkness; and effects of far-red LED on circadian rhythm and ERG in rodents are lacking.

In the present study, we tested the hypotheses that there are long wavelength red LED lights which can be perceived by human eyes, but not by rodent eyes due to the lack of red cones (L-cones) in these animals; thus, use of these long wavelength LED at night or dark phase would not interfere with their behaviors and circadian rhythms. We systemically examined effects of a spectrum of long wavelength LED lights (peak at 642, 666, 741, 850, and 940 nm) on locomotor activity, body temperature, and cardiovascular circadian rhythms and ERG in C57BL/6J mice and Wistar Han rats.

## Materials and methods

### Ethics statement

All procedures performed on animals in this study were in accordance with regulations and established guidelines and were reviewed and approved by Pfizer Institutional Animal Care and Use Committee. Pfizer animal care facility that supported this work is fully accredited by AAALAC International.

### Animals for circadian study

Male C57BL/6J mice (strain # 664, n = 4, age 10−12 weeks) and Wistar Han rats (strain # 273, n = 15, age 10−11 weeks) were obtained from Jackson Lab (Bar Harbor, ME) and Charles River Lab (Raleigh, NC), respectively. Animals were free of specific pathogens and singly housed in Innovive® cages (Innovive, Billerica, MA) on a static rack on Alpha-Dri® (Shepherd Specialty Paper, Richland, MI) bedding and maintained at 22 ± 1°C, 30−70% relative humidity, and 12:12-h white light:dark cycle (LD; lights on, 6 AM – 6 PM; light off, 6 PM – 6 AM). Mice and rats were fed Lab Diet #5053 chow (Purina, St. Louis, MO) and water *ad libitum*. Animals were euthanized by CO_2_ after completion of the study.

### Surgical implantation of telemetry devices

After one week acclimation in the study facility from receipt of the animals, mice and rats were implanted intraperitoneally with TA-F10 and PA-C40 telemeters (Data Sciences International [DSI], St. Paul, MN), respectively.

Aseptic technique was strictly applied during all surgical procedures. Animals were anesthetized using 2–3% isoflurane in oxygen. Pre-emptive 5 mg/kg carprofen was administered subcutaneously to reduce post-operative pain. The surgical site was clipped free of hair and prepared for aseptic surgery using alternating wipes of appropriate surgical scrub, 70% ethanol, and sterile saline. Ophthalmic ointment was instilled in the animal’s eyes to prevent corneal drying. Anesthetic depth, respiratory rate and pattern were periodically monitored. Depth of anesthesia was assessed by loss of movement, loss of blink reflex, and lack of response to toe pinch. Toe pinch was monitored at 10–15 minute intervals through the surgery procedure. The body temperature of mice was maintained by using a circulating-water heating pad.

TA-F10 telemeters were implanted in mice. Briefly, after a small skin and laparotomy incision (approximately 2 cm in length) was made along the abdominal midline, a telemetry device was wetted with sterile saline and inserted into the peritoneal cavity. The laparotomy incision and subcuticular layers were closed with sterile absorbable suture. Surgical adhesive was also used to aid in skin closure.

PA-C40 telemeters were implanted in rats. Briefly, an abdominal midline incision was made extending to slightly above the xiphoid cartilage. Abdominal wall and viscera were covered with saline moistened gauze. Intestines were retracted to expose and isolate the descending aorta. A circular purse string suture was placed in the aorta tunica using a non-absorbable suture. Two clamps were used to occlude the aorta, one on the aorta near the renal artery and the other at the common iliac bifurcation. The aorta was pierced in the center of purse string using a curved needle and the catheter was guided into the vessel retrograde with the tip resting distal to the renal artery. The purse string suture was quickly tightened down around the catheter to seal the juncture. Clamps were removed to allow the timely reperfusion of distal body parts. The telemetry device with suture ribs was then fixed to the abdominal wall with non-absorbable suture. The peritoneal cavity was then irrigated with warm, sterile saline and the intestines put back in place. Incisions and subcuticular layers were closed with absorbable suture.

Opioid analgesic (a dose of sustained release formulation of buprenorphine HCl at 1 mg/kg SC) was provided for a minimal coverage of 72 hours post-surgery. The animals were placed in warm and clean cages for recovery. Once ambulatory they were returned to the holding room and assessed daily for 7–10 days until the incision was fully healed.

### Light source

**Fig 1:**
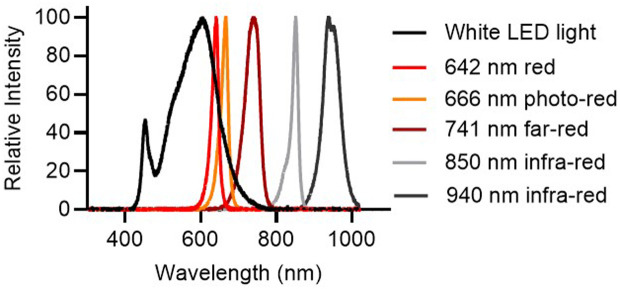
Light spectrum. White LED light, 642 ± 10 nm (peak wavelength± peak half width) red, 666 ± 11 nm photo-red, 741 ± 23 nm far-red, and 940 ± 26 nm infra-red LED lights were used in circadian experiments at approximately 50 µW/cm^2^ inside of cages (i.e., 172, 60, 25, 1, and 0 lux, respectively). The 850 ± 9 nm infra-red LED was used in ERG experiments.

The LED lights used in this study were mostly purchased from LEDSupply (Randolph, VT). The LEDs used for the circadian study were mounted on LuxStrip (Cat# 9008, LEDSupply), a high-power LED strip lighting. Each LuxStrip contains 6 high power LEDs (i.e., neutral white LED: Part# CREEXPE2-WHT; red LED: Part# CREEXPE2-RED; photo-red (also called as deep-red) LED: Part# CREEXPE-DRD; far-red LED: Part# CREEXPE-FRD; and 850 nm infra-red LED: Part# OSGD-IR-850; DC forward current: 700–1000 mA for all LEDs). Ultra-covert IR-flood infra-red illuminator (940 nm) (Night Vision Experts, Toronto, Canada) was used in the circadian experiments. The spectral power distribution of LED lights used in this study (Fig 1) was obtained by using a compact spectrometer (range, 200–1000 nm; model# CCS200, ThorLabs, Dachau, Germany). Cages were placed on telemetry receivers on a static rack and were positioned to ensure the maximal uniformity of lighting. An optical power and energy meter (model# PM 100D, ThorLabs) and a light meter (model# LM631A, Amprobe, Everett, WA) were used to obtain the irradiance and illuminance of source lights. For the circadian study, the source lights were adjusted to have an irradiance approximately 50 µW/cm^2^ inside of the cages at rodent eye levels. The Innovive cages used in the study were made of clear transparent plastic and the irradiance of source lights were maintained consistent for all different lights used in the circadian study.

Circadian study was initiated 4 weeks after surgery, when animals had restored their pre-surgery body weights. To prevent potential light contamination and circadian interference, animals were housed in a room that was light-tight to external lights, and light indicators on instrument panels within the study room were covered with foil.

Various lighting paradigms were used to assess the effects of different source lights on circadian rhythms in animals. At the start of the experiment, animals were maintained under standard 12:12-h white light:dark (LD) cycle until baseline circadian data were collected for approximately 2 weeks, then the following conditions were tested. First, to determine whether a test light would entrain rodent circadian rhythms, mice were placed under a delayed 12:12h light:dark cycle for far-red and photo-red LED, respectively, for one week each LED light, followed by one week under constant darkness (DD). Under DD condition, a night vision goggle (Cat# ATN NVM14, American Technologies Network Corporation, South San Francisco, CA) was used to facilitate animal husbandry and health monitoring. In rat circadian study, an advanced 12:12h light:dark cycle was used for photo-red and far-red light, respectively, after one week at DD condition. Second, to compare different long wavelength light’s effect on the free-running circadian period vs constant darkness condition, animals were placed under a constant light condition for each test light for approximately one week. Third, to mimic a study procedure, animals were exposed to a 2-hour test light during the dark phase of a standard light cycle. Lastly, animals were housed in a room where the dark phase was replaced by a 12-hour test light to evaluate its interference in the circadian profile vs those under standard light cycle condition.

### Circadian data acquisition and analyses

DSI Dataquest A.R.T. system (version 4.36) was used to collect circadian data. In the mouse circadian study, core body temperature data were captured continuously at 250 Hz sampling rate from individual animals. The locomotor activity data was generated by the Data Exchange Matrix in the Dataquest A.R.T. system according to the changes in telemetry signal strength received by the receiver antennas when the animal moved around in its cage. The activity data provided by the Dataquest A.R.T. software is a relative measure of locomotor activity, which is dependent on both distance and speed of animal’s movement. In the rat circadian study, arterial blood pressure, heart rate, and locomotor activity data were collected continuously. Arterial blood pressure was collected at 500 Hz sampling rate and heart rate (HR) was derived from the blood pressure waveform. Mean arterial pressure (MAP), heart rate, and locomotor activity measurements were logged continuously as 1-minute means.

**Fig 2:**
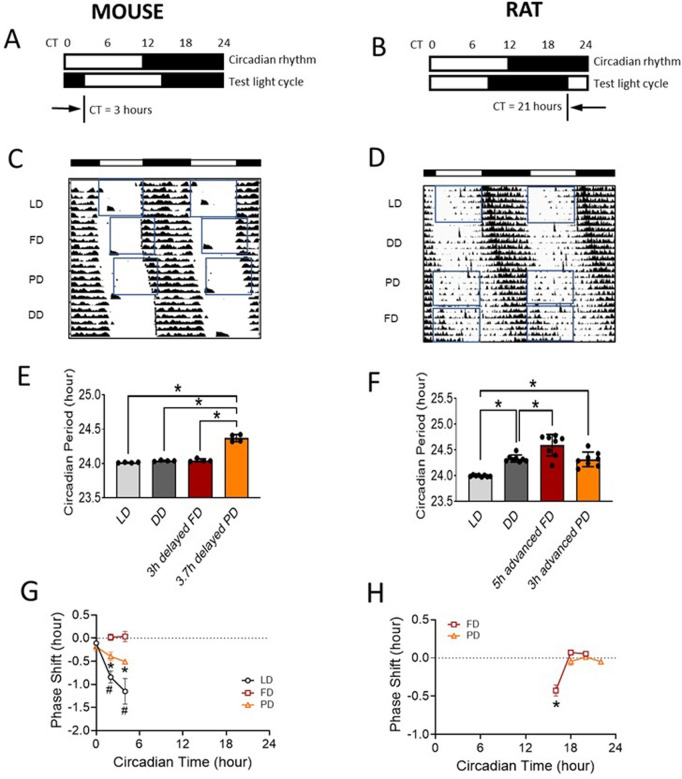
Circadian entrainment or phase shift. **A)** Light protocol for mouse entrainment experiment. Delayed 12:12h light:dark cycle (dusk signal) was used to entrain mice or to observe phase shift. **B)** Light protocol for rat entrainment experiment. Advanced 12:12h light/dark cycle (dawn signal) was used to entrain rats or to observe phase shift. **C)** Double plotted mouse body temperature (TEMP) actogram (i.e., 2 consecutive days plotted next to each other). **D)** Double plotted rat heart rate (HR) actogram. **E)** Circadian period obtained from onset of TEMP circadian rhythm in mice. **F)** Circadian period obtained from onset of HR circadian rhythm in rats. **G)** 12h light-induced TEMP circadian phase responses (partial phase response curve) in mice (details see S5 Fig). Daily phase shift was calculated from TEMP onset and subtracted by daily phase shift in DD. Data were then binned into 2-hour circadian time (CT) intervals. **H)** 12h light-induced HR circadian phase responses (partial phase response curve) in rats (details see S6 Fig). White bar: 12h light-on phase; black bar: 12h light-off phase. Open rectangle = light-on period. CT 0-12 = subjective light phase; CT 12-24 = subjective dark phase. LD = 12:12h white light:dark; FD = 12:12h far-red light:dark; PD = 12:12h photo-red light:dark; DD = 24h constant darkness. Data: mean ± SD. N = 4 mice/8 rats. *Significantly different between groups (E&F), or different from FD and zero **(G)**, or different from PD and zero **(H)** (P < 0.05, ANOVA). #Significantly different from PD **(G)** (P < 0.05, ANOVA).

Actograms and circadian robustness were generated by using ClockLab 6.1.05 (Actimetrics, Wilmette, IL). Circadian parameters (amplitude, period, acrophase, and mesor) were calculated using MatLab 7.11.0 (R2010b) (Natick, MA) by cosine fitting on 5 consecutive days 5-min means dataset obtained under each lighting condition. The circadian phase responses induced by 12-hour far-red and photo-red lights were calculated from delayed and advanced 12:12h light:dark cycles in each animal. The circadian time (CT) was determined by the onset of circadian rhythm with respective to the light cycles. The circadian phase shift was obtained as day-to-day onset phase shift, subtracting the phase shift caused by free-running circadian rhythm under DD condition for individual animals (Fig 2G, 2H).

### Full-field electroretinogram (ERG) recording

Male C57BL/6J mice (n = 7; age 14–16 weeks; Jackson Labs, #664) and male Wistar Han rats (n = 6; age 10–12 weeks; Charles River Labs, #273) were used in the ERG testing. All animals were exposed to a standard LD cycle, with free access to food and water. ERG was tested during the daytime (8 AM to 4 PM). The UTAS BigShot Visual Electrodiagnostic System (Model# E-3000, LKC Technologies, Gaithersburg, MD) was used to evoke and acquire the ERG signals (high-pass filtered at 0.3 Hz and low-pass filtered at 500 Hz). ERG signals were collected from both eyes of the animal simultaneously at 2K Hz sampling rate from start of the flash light for 255 ms sweep length. The high-power LED lights used in the ERG study were mounted on a LuxDrive Star Board Configuration (triple LEDs mounted upwards). The LEDs used in the ERG study were the same as those used in the circadian study, except for the infra-red LEDs, and their spectral power distribution is shown in Fig 1. These customized LEDs were placed over the original LEDs to evoke ERG signals. An Arduino Uno micro-board (Vilros, Lakewood, NJ) was used to control the timing of the LED flashes (S1 Fig). The flash was initiated by the Trigger Out signal from the LKC UTAS ERG system. An enhancement N-channel logic level MOSFET (model #NTE2980, Wholesale Electronics, Inc., Mitchell, SD) was used to turn on and off LEDs. High power potentiometers were used to control the intensity of LED flashes. The LED intensity was measured at the level of the animal’s eye and calibrated using the optical power and energy meter and the light meter described above. The LEDs spectrum was confirmed using the spectrometer (CCS200) and the duration of LED flashes (i.e., 5 ms) was confirmed using a digital storage oscilloscope (model # TBS1052C, Tektronix, Beaverton, OR).

Full-field ERG was performed as previously reported [36]. Briefly, animals were dark-adapted for approximately 2 h and then anesthetized with 1.5% to 2.5% isoflurane in oxygen. Body temperature was maintained using a circulating water heating pad (T/Pump, Gaymar, Orchard Park, NY). Tropicamide (1%; Mydriacyl, Bausch and Lomb, Tampa, FL) was applied to the eyes to induce pupil dilation. ERG lens electrodes (Medical Workshop, Groningen, Holland) were placed onto the surfaces of both eyes by using 0.25% hypromellose (Hub Pharmaceutical, Rancho Cucamonga, CA) and sterile saline as a coupling agent. A platinum needle reference electrode (Natus Neurology, West Warwick, RI) was inserted subcutaneously between eyes on the forehead scalp; and the ground was placed on the tail.

Dark-adapted ERG responses were measured for all LED lights at 0.42, 4.2, 42, 420, and 4200 µW/cm^2^ (irradiance at the level of animal’s eyes), in ascending order, after the dark-adaptation. The corresponding illuminance for white LED light was approximately 1, 10, 100, 1000, and 10000 lux, respectively. At each light intensity (irradiance) level, infra-red, far-red, photo-red, red, and white LED light flashes were delivered 10 times each LED at 0.1 Hz to obtain mean ERG signals. At the end, 4.2 µW/cm^2^ (10 lux) white LED flashes were repeated to ensure that ERG signals were consistent over the time course.

After dark-adapted ERG responses testing, the animals were exposed to white LED light at 42 µW/cm^2^ (100 lux) for 10 min for light adaptation prior to light-adapted ERG responses testing. The light-adapted ERG responses were tested against a bright background when the rod system was desensitized by the light. Light-adapted ERG responses testing was performed for all LED lights at 42, 420, and 4200 µW/cm^2^ (high intensity levels – irradiance) in 5 ms flashes, from low to high intensity respectively. LED light flashes were delivered 10 times each at 1 Hz to obtain mean ERG signals.

ERG waveforms were analyzed by using manufacturer-provided software under the guidelines of the International Society for Clinical Electrophysiology of Vision [37]. The a-wave amplitude was measured from baseline to trough, and latency was measured between stimulus artifact and a–wave trough. The b-wave amplitude was measured from a-wave trough to b-wave peak, and b-wave latency was measured from stimulus artifact to b-wave peak.

After completion of the study, additional far-red LEDs with peak wavelength at 750, 760, 770, and 780 nm (cat# LED750L, LED760L, LED770L, and LED780L, respectively) were obtained from ThorLabs for visibility confirmation and calculation of projected ERG and circadian responses.

### Statistical analysis

Circadian data statistical analysis was performed by using a mixed linear model (PROC MIXED) in SAS version 9.4 (SAS Institute, Cary, NC), and the Fisher least significant difference post-hoc test was used to analyze differences between light cycles. The model included light cycle and cage change status as fixed categorical factors and animal age as a covariate. Animal ID was included in the RANDOM statement of the MIXED model. Comparisons between light cycles were generated based on model means. A P-value of less than 0.05 indicates a statistically significant difference between light cycles. For ERG data, light type was included as categorical factor and light intensity as repeated measures. Data are primarily presented as mean ± SD in figures and tables, except for the data listed in S1 ands S2 Tables, where least squares means and standard errors are used to account for observed age-related effect on circadian parameters (S2 and S3 Figs).

## Results

Under different lighting conditions, mice and rats displayed similar circadian patterns in core body temperature, heart rate, blood pressure, and locomotor activity.

### Circadian entrainment or phase shift

To determine whether the long wavelength LED lights would entrain rodent circadian rhythms, mice were placed in a delayed 12:12h test light:dark cycle and rats were placed in an advanced 12:12h test light:dark cycle. During normal light-induced entrainment process, prolonged circadian periods are associated with circadian onset phase delays (S5 Fig), and shortened circadian periods are associated with circadian onset phase advances. Thus, circadian phase shifts and circadian periods under those conditions were examined and compared with those under DD and LD conditions.

When mice were placed under a 3-hour-delayed 12:12h far-red:dark (FD) cycle, no significant circadian phase shift was observed vs DD condition (Fig 2A and 2C). The body temperature circadian period under the delayed FD cycle (24.05 ± 0.02 mean ± SD hr) was not different from those under DD condition (24.04 ± 0.01 hr) (Fig 2E). However, when mice were placed under approximately 3.7-hour-delayed 12:12h photo-red:dark (PD) cycle (accounted circadian phase shift during previous FD light cycle), a significant circadian phase delay was observed vs DD condition (Fig 2C). The circadian period under delayed PD cycle (24.37 ± 0.05 hr) was significantly prolonged when compared with those under DD and LD conditions (P < 0.05, ANOVA) (Fig 2E), indicating a significant circadian onset delay vs DD and LD. In addition, FD and PD did not induce significant changes in body temperature circadian robustness and amplitude vs DD condition (S1 Table).

In the rat circadian study, an advanced 12:12h light:dark cycle was used to evaluate heart rate (HR) circadian phase responses (Fig 2B). After one week at DD condition, animals’ circadian rhythm was free-running under the control of their endogenous circadian clock (Fig 2D). Then rats were exposed to an approximately 3-hour advanced 12:12h photo-red:dark cycle for 7 days. The circadian phase shifts during the previous cycles were included in the calculations for the advanced phases applied. It was surprising to find that the 3-hour advanced PD cycle did not cause phase advances in rats vs DD condition. The heart rate circadian period under the advanced PD cycle (24.32 ± 0.14 mean ± SD hr) was not different from those under DD condition (24.33 ± 0.07 hr) (Fig 2F), indicating no significant onset phase shift vs DD. However, the heart rate circadian amplitude, mesor, and robustness were all significantly reduced under advanced PD cycle vs DD and LD conditions (P < 0.05, Mixed Linear Model performed using PROC MIXED procedure in SAS, included all rat circadian data) (S2 Table), indicating a disruption to circadian rhythms. Moreover, when rats were placed under an approximately 5-hour advanced 12:12h far-red:dark cycle for 7 days, animals demonstrated a significant onset phase delay vs DD condition (Fig 2D). The heart rate circadian period under 5-hour advanced FD cycle (24.59 ± 0.21 hr) was significantly greater than those under DD and LD conditions (P < 0.05, ANOVA) (Fig 2F), indicating a significant onset phase delay vs DD and LD. And the heart rate circadian amplitude and robustness under the advanced FD cycle was significantly reduced vs DD and LD conditions as well (P < 0.05, Mixed Linear Model, included all rat circadian data) (S2 Table).

A partial phase response curve was obtained from the entrainment protocol (see S5 and S6 Figs for details). In mice, 12-h far-red did not induce any phase delay at CT 2 and 4 hours, whereas photo-red and white light did cause significant phase delays at corresponding CT hours vs DD condition (P < 0.05, ANOVA, DD: as zero phase shift) (Fig 2G). Further, the phase delays induced by the 12-h photo-red light were significantly smaller than those by the 12-h white light (P < 0.05, ANOVA). In rats, both 12-h far-red and photo-red lights did not induce phase advances at CT 18–22 hours (P < 0.05, ANOVA) (Fig 2H). Nevertheless, 12-h far-red light induced significant phase delays at CT 16 hours (P < 0.05, ANOVA).

### Free-run circadian period under constant light (Aschoff effect)

The Aschoff’s first rule states that the endogenous free-run circadian period (*tau*) observed in DD will lengthen for nocturnal animals when they are exposed to constant light and the effects of constant light are intensity dependent with brighter light enhancing these effects. The Aschoff’s second rule states that under constant bright light, activity time (*alpha*) decreases for nocturnal animals and the duration of daily activity in constant light conditions decreases with increasing light intensity.

To quantify the effect of different long wavelength LED lights on the free-run circadian period vs DD condition according to the Ashoff’s rules animals were placed under a constant light condition for each test light for approximately one week.

**Fig 3:**
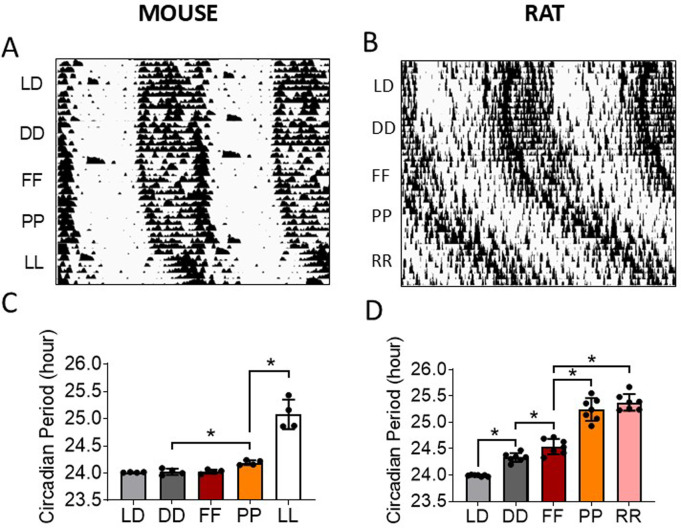
Free-running circadian rhythm under constant light condition. **A)** Double plotted mouse body temperature (TEMP) actogram. **B)** Double plotted rat heart rate (HR) actogram. **C)** Circadian period obtained from onset of mouse TEMP circadian rhythm. **D)** Circadian period obtained from onset of rat HR circadian rhythm. LD = 12:12h light:dark; DD = constant darkness; FF = constant far-red; PP = constant photo-red; RR = constant red; LL = constant white light. Data: mean ± SD. N = 4 mice/ 8 rats. *Significantly different between light conditions (P < 0.05, ANOVA).

In C57BL/6J mice, free-run body temperature circadian period was significantly prolonged under constant photo-red (PP) and constant white light (LL) vs DD condition (P < 0.05, ANOVA) (Fig 3A and 3C). The endogenous free-run body temperature circadian periods were 24.03 ± 0.06, 24.03 ± 0.04, 24.19 ± 0.04, and 25.08 ± 0.27 (mean ± SD) hr under DD, FF, PP, and LL conditions, respectively (i.e., periods under DD and FF < PP < LL, P < 0.05, ANOVA) (Fig 3C). Mice under constant far-red light (FF) condition did not prolong circadian period when compared to DD condition. In addition, the activity time (alpha) were 12.08 ± 0.43, 12.76 ± 0.58, 12.19 ± 0.65, and 10.42 ± 0.63 (mean ± SD) hr under DD, FF, PP, and LL conditions, respectively. The active time under LL condition was significantly decreased compared with DD condition, and the active time under PP cycle was significantly shorter than those under FF cycle (P < 0.05, ANOVA). Body temperature circadian amplitude and robustness in mice were significantly reduced under LL (P < 0.05, Mixed Linear Model, included all mice circadian data), but not under FF and PP, vs DD condition (S1 Table).

In Wistar Han rats, free-run HR circadian period was prolonged under FF, PP, and constant red (RR) conditions vs DD (P < 0.05, ANOVA) (Fig 3B and 3D). The endogenous free-run HR circadian periods were 24.33 ± 0.08, 24.54 ± 0.15, 25.24 ± 0.21, and 25.38 ± 0.16 (mean ± SD) hr under DD, FF, PP, and RR conditions, respectively (i.e., periods under DD < FF < PP and RR, P < 0.05, ANOVA) (Fig 3D). Interestingly, it was noted that FF prolonged free-run circadian period in Wistar Han rats (P < 0.05, ANOVA), but not in C57BL/6J mice (Fig 3C and 3D).

The lengthening of free-run circadian period under constant FF, PP, and RR conditions in rats (0.21 ± 0.13, 0.91 ± 0.15, and 1.0 ± 0.14 mean ± SD hr, respectively) was significantly greater than those under constant FF and PP in mice (0 ± 0.02 and 0.17 ± 0.07 hr, respectively) (FF in mice < PP in mice and FF in rat < PP and RR in rat, P < 0.05, ANOVA).

### Two-hour red light exposure in dark phase

To mimic a study procedure, animals were exposed to 2-h test light during the dark phase of a standard LD light cycle. Circadian profiles under this test protocol were then examined and compared to those under the LD condition for mice and rats.

**Fig 4:**
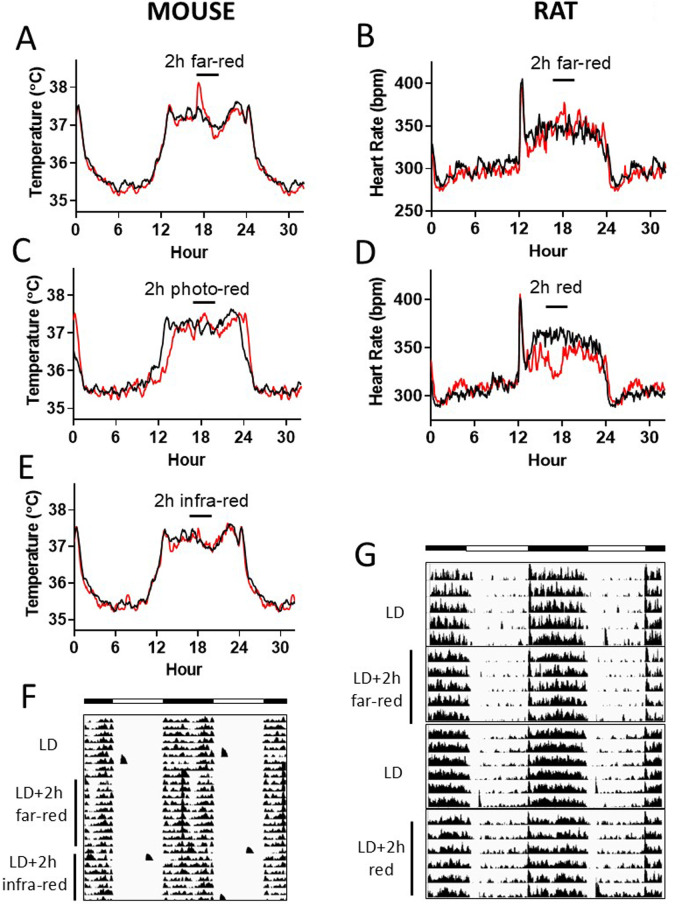
Two-hour test light in the dark phase. A & C & E) Mouse body temperature (TEMP) profiles. B&D) Rat heart rate (HR) profiles. **F)** Double plotted mouse TEMP actogram. **G)** Double plotted rat HR actogram. White bar: light phase; black bar: dark phase. Black line: standard light/dark cycle; red line: 2h-test light in the dark phase of a standard light/dark cycle. Test lights were placed at ZT 16-18 daily for approximately one week as indicated. ZT 0-12 = light phase; ZT 12-24 = dark phase. LD = 12:12h white light:dark. Profile data: means. N = 4 mice/ 7 rats.

In C57BL/6J mice, exposure of 2-h far-red light in the dark phase (LD + 2hF) induced a dramatic biphasic transient response in body temperature, i.e., body temperature increased when the far-red light started, then it decreased after an hour of the far-red light exposure (Fig 4A and 4F). The amplitude of mouse body temperature biphasic wave induced by 2-hour far-red light was significantly different from LD, LD + 2h photo-red, and LD + 2h infra-red conditions (P < 0.05, ANOVA) (S7A Fig). And exposure of 2-h photo-red light in the dark phase (LD + 2hP) delayed body temperature circadian onset (P < 0.05, ANOVA) (Fig 4C, S7F Fig). However, exposure of 2-h infra-red (λ_p_ = 940 nm) light in the dark phase (LD + 2hI) did not cause any noticeable change in body temperature circadian rhythm (Fig 2E, 2F, S7 Fig).

In Wistar Han rats, LD + 2hF light cycle induced a similar transient response in HR circadian profile (Fig 4B). The amplitude of rat heart rate biphasic or monophase waves induced by 2-hour infra-red and red lights were significantly different from LD condition (P < 0.05, paired t-test) (S7B and S7C Fig). Exposure of 2-h red light in the dark phase (LD + 2hR) dramatically decreased HR during the red-light exposure (P < 0.05, paired t-test) (Fig 4D and 4G, S7G Fig). HR circadian amplitude and robustness were significantly decreased under LD + 2hF and LD + 2hR conditions vs LD (P < 0.05, Mixed Linear Model, included all rat circadian data) (S2 Table).

Locomotor activity circadian data demonstrated similar transient responses to LD + 2hF light cycle in C57BL/6J mice and Wistar Han rats (S4 Fig).

### Twelve-hour red light in whole dark phase

To minimize the effect of sudden exposure to 2-h test light during the dark phase, animals were housed in a room where the dark phase was replaced by 12-hour test light. We hypothesized that having the test LED light on during the whole dark phase would mitigate the dramatic transient responses observed with the sudden exposure to light in the dark phase.

**Fig 5:**
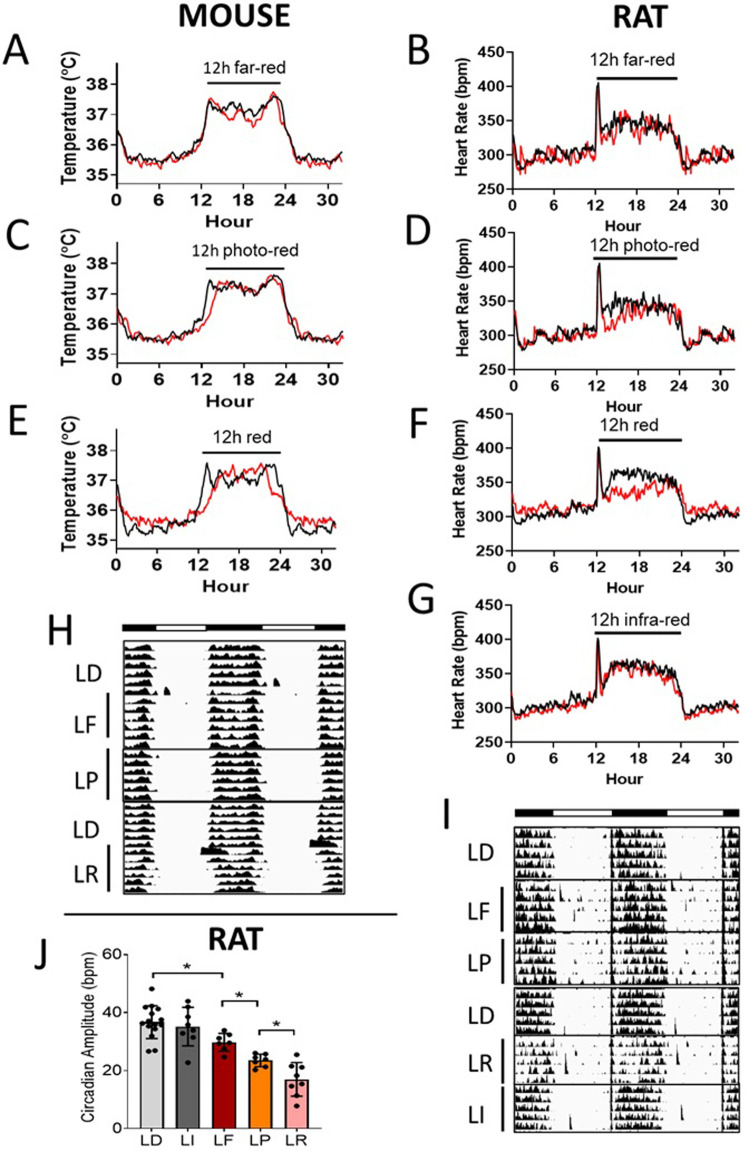
Twelve-hour test light in the whole dark phase. A & C & E) Mouse body temperature (TEMP) profile. B & D & F & G) Rat heart rate (HR) profiles. **H)** Double plotted mouse TEMP actograms. **I)** Double plotted rat HR actograms. **J)** Rat HR circadian amplitude (mean ± SD). White bar: light phase; black bar: dark phase. Black line: standard light/dark cycle; red line: 12h-test light in the whole dark phase of a standard light/dark cycle. Test lights were placed at ZT 12-24 daily for approximately one week as indicated. ZT 0-12 = light phase; ZT 12-24 = dark phase. LD = 12:12h white light:dark; LI = 12:12h white light:infra-red; LF = 12:12h white light:far-red; LP = 12:12h white light:photo-red; LR = 12:12h white light:red light. Profile data: means. N = 4 mice/ 7 rats. *P < 0.05, ANOVA.

In C57BL/6J mice, 12-h far-red light in the whole dark phase (LF) decreased body temperature in the middle of the dark phase (P < 0.05, ANOVA) (Fig 5A and 5H, S8A Fig) and the dramatic transient biphasic response disappeared as expected (Fig 5A and 5H). No significant circadian phase shift was noted when far-red light was used. However, 12-h photo-red light in the whole dark phase (LP) delayed body temperature circadian onset vs LD condition (P < 0.05, ANOVA) (Fig 5C and 5H, S8E Fig). And 12-h red light in the whole dark phase (LR) dramatically altered body temperature circadian profile and significantly reduced the active time (alpha) which is similar to what was observed under the constant light condition (Fig 5E and 5H). Under LR condition, body temperature circadian acrophase was significantly delayed (P < 0.05, Mixed Linear Model) and circadian robustness was significantly reduced (P < 0.05, Mixed Linear Model) as well (S1 Table).

In Wistar Han rats, heart rate circadian amplitude was significantly decreased under LF, LP, and LR cycles vs LD condition (P < 0.05, ANOVA) (Fig 5J). The magnitude of HR circadian amplitude decreases under LF, LP, and LR conditions were 15, 31, and 41%, respectively, vs LD condition (i.e., LF < LP < LR, P < 0.05, ANOVA). HR circadian robustness under LP condition was significantly smaller vs LD condition as well (P < 0.05, Mixed Linear Model, included all rat circadian data) (S2 Table). The circadian robustness under LF and LR condition were not captured due to unexpected events. However, 12-h infra-red (λ_p_ = 940 nm) light in the whole dark phase (LI) did not cause any noticeable change in heart rate circadian rhythm in Wistar Han rats (Fig 5G and 5I, S8 Fig).

Locomotor activity circadian data demonstrated similar small responses to the LF light cycle in C57BL/6J mice and Wistar Han rats (S4 Fig).

### Dark-adapted ERG responses

In order to understand the mechanisms involved in the regulation of circadian rhythms by LED lights in rodents, we completed dark-adapted and light-adapted ERG recordings in C57BL/6J mice and Wistar Han rats.

**Fig 6:**
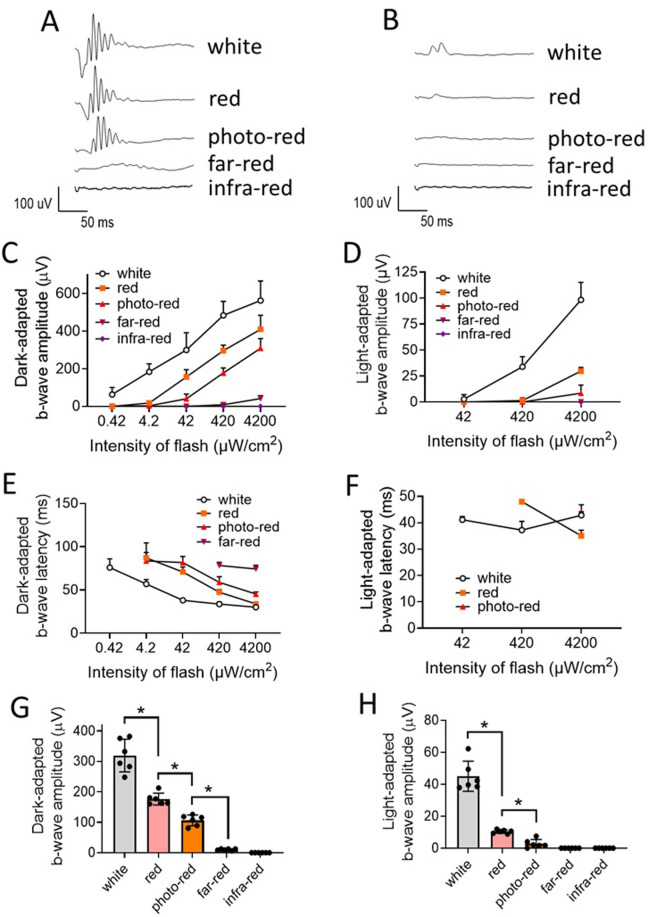
Dark-adapted and light-adapted electroretinogram (ERG) responses in C57BL/6J mice. **A)** Representative dark-adapted ERG traces recorded after LED light flashes at 4200 µW/cm^2^ for 5 ms. Animals were adapted to the darkness for at least 2 hours prior to the dark-adapted ERG recording. **B)** Representative light-adapted ERG traces recorded after LED light flashes at 4200 µW/cm^2^ for 5 ms. Animals were exposed to white LED light at 42 µW/cm^2^ for 10 min for light adaptation prior to the testing. **C)** Dark-adapted b-wave amplitude. **D)** Light-adapted b-wave amplitude. **E)** Dark-adapted b-wave latency. **F)** Light-adapted b-wave latency. **G)** Mean dark-adapted ERG b-wave amplitude from all levels of stimuli. **H)** Mean light-adapted ERG b-wave amplitude from all levels of stimuli. Data: mean ± SD. N = 7. *Significantly different between groups (P < 0.05, ANOVA).

In C57BL/6J mice, dark-adapted b-wave amplitude was significantly different between white, red, photo-red, and far-red LED lights (i.e., white > red > photo-red > far-red, P < 0.05, ANOVA) (Fig 6A and 6G). The mean dark-adapted b-wave amplitude obtained from 5 stimulus intensity levels (i.e., 0.42, 4.2, 42, 420, and 4200 µW/cm^2^) for white, red, photo-red, and far-red LED lights were 291 ± 89, 162 ± 41, 97 ± 30, and 9 ± 4 µV (mean ± SD), respectively. The dark-adapted b-wave latency were significantly different between white, red, photo-red, and far-red light (i.e., white < red < photo-red < far-red, P < 0.05, ANOVA) at 420 µW/cm^2^ (Fig 6E, S3 Table).

**Fig 7:**
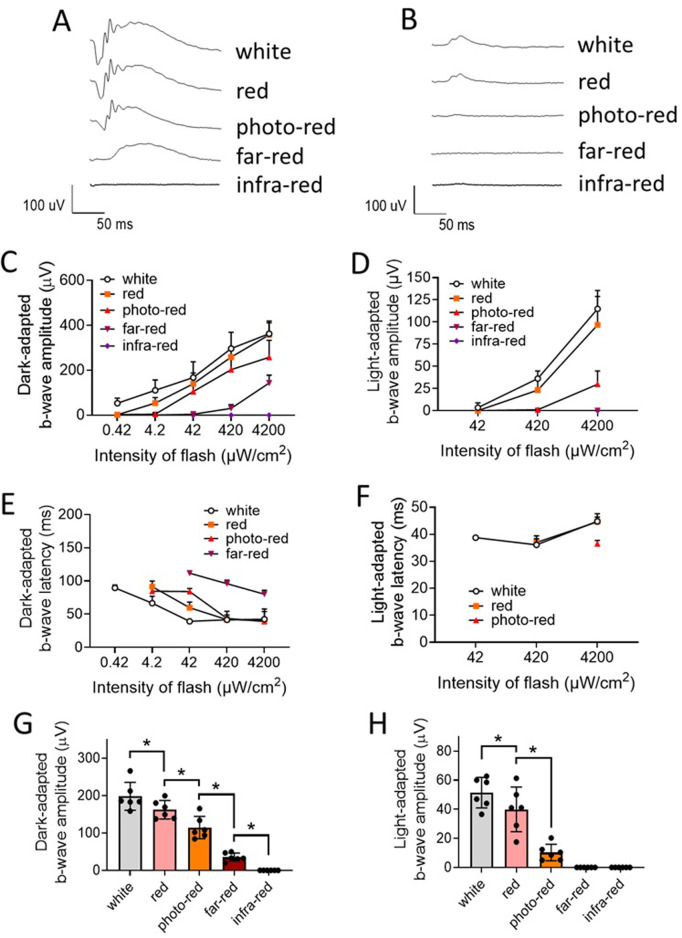
Dark-adapted and light-adapted electroretinogram (ERG) responses in Wistar Han rats. **A)** Representative dark-adapted ERG traces recorded after LED light flashes at 4200 µW/cm^2^ for 5 ms. Animals were adapted to the darkness for at least 2 hours prior to the dark-adapted ERG recording. **B)** Representative light-adapted ERG traces recorded after LED light flashes at 4200 µW/cm^2^ for 5 ms. Animals were exposed to white LED light at 42 µW/cm^2^ for 10 min for light adaptation prior to the testing. **C)** Dark-adapted b-wave amplitude. **D)** Light-adapted b-wave amplitude. **E)** Dark-adapted b-wave latency. **F)** Light-adapted b-wave latency. **G)** Mean dark-adapted ERG b-wave amplitude from all levels of stimuli. **H)** Mean light-adapted ERG b-wave amplitude from all levels of stimuli. Data: mean ± SD. N = 6. *Significant different between groups (P < 0.05, ANOVA).

In Wistar Han rats, similar dark-adapted ERG results were obtained. Dark-adapted b-wave amplitudes from rats were significantly different between white, red, photo-red, and far-red LED lights (i.e., white > red > photo-red > far-red, P < 0.05, ANOVA) (Fig 7A and 7G). The mean dark-adapted b-wave amplitudes obtained from 5 stimulus intensity levels (i.e., 0.42, 4.2, 42, 420, and 4200 µW/cm^2^) for white, red, photo-red, and far-red LED lights were 198 ± 37, 162 ± 25, 115 ± 30, and 35 ± 11 µV (mean ± SD), respectively. The dark-adapted b-wave latency was significantly different between white, red, photo-red, and far-red lights (i.e., white < red < photo-red < far-red, P < 0.05, ANOVA) at 42 µW/cm^2^ (Fig 7E, S4 Table). Interestingly, it was noted that the far-red light elicited greater dark-adapted ERG responses in Wistar Han rats than in C57BL/6J mice (P < 0.05, T-test) (Figs 6A, 6G, 7A and 7G, S3 and S4 Tables).

No dark-adapted ECG signal was detected in both mice and rats with infra-red LED (λ_p_ = 850 nm) light at all intensities tested (Figs 6 and 7, S3 and S4 Tables).

### Light-adapted ERG responses

In C57BL/6J mice, light-adapted b-wave amplitudes were significantly different between white, red, and photo-red LED lights (i.e., white > red > photo-red, P < 0.05, ANOVA) (Fig 6B and 6H). The mean light-adapted b-wave amplitudes obtained from 3 stimulus intensity levels (i.e., 42, 420, and 4200 µW/cm^2^) for white, red, and photo-red LED lights were 42 ± 12, 10 ± 1.1, and 2.4 ± 2.6 µV (mean ± SD), respectively.

In Wistar Han rats, similar light-adapted ERG results were obtained. Light-adapted b-wave amplitudes from rats were significantly different between white, red, and photo-red LED lights (i.e., white > red > photo-red, P < 0.05, ANOVA) (Fig 7B and 7H). The mean light-adapted b-wave amplitudes obtained from 3 stimulus intensity levels (i.e., 42, 420, and 4200 µW/cm^2^) for white, red, and photo-red LED lights were 51 ± 10, 40 ± 15, and 10 ± 5.7 µV (mean ± SD), respectively.

No light-adapted ERG signal was detected from both mice and rats with both far-red and infra-red LED lights at all intensities tested (Figs 6 and 7, S3 and S4 Tables).

## Discussion

In this report, we first demonstrate that all visible long wavelength red lights, including the far-red LED light with a peak at 741 nm, induce significant alterations in circadian rhythms such as phase delays, and dark-adapted ERG responses in mice and rats. These data are complementary to a previous report demonstrating that rats retain degraded object discrimination capacity under 729 nm far-red LED illumination [35] and suggest that mice and rats indeed partially reserve both image-forming (form vision) and non-image forming visual (circadian regulation) capacities under far-red light condition. In contrary, invisible infra-red LED lights do not induce detectable changes in circadian rhythm or any dark-adapted ERG responses in these animals. Thus, our findings suggest that mice and rats are truly sensitive to exposures of all visible red lights during the dark phase, possibly mediated through the rod photoreceptors.

The effects of red light on circadian rhythms in rodent have long been reported [30,32–34,38–40] and the magnitude of circadian responses to the red light is light-intensity dependent as well. In 1973, it was demonstrated that the red light (λ = 660 ± 19 nm, peak wavelength ± peak half width) entrained body temperature circadian rhythm in all 8 (100%) Charles River CD rats at 30 µW/cm^2^, while at 10 µW/cm^2^ only 50% of the rats were entrained [39]. In another study, albino rats that were free-running under constant darkness were exposed to 2-h of filtered red light (λ > 650 nm and intensity at 4.7 µW/cm2) near the beginning (dust signals) or the end (dawn signals) of their active 12-h period of running. Red dawn signals advanced and red dusk signals delayed the onset of running on subsequent days and animals’ activity and ovulation rhythms were eventually entrained to the red light [38]. In early studies, filtered red light sources were commonly used and the purity of the red light was a concern [38,39]. Recently, LED light sources were often used to circumvent the non-red light contamination issue [32,33,35].

In this study, the 741 nm far-red LED was shown to induce circadian phase delay and prolong free-run circadian period in Wistar Han rats at 50 µW/cm^2^. Rats that were free-run under DD were exposed to 12-h photo-red and 12-h far-red LED lights, respectively, at the end of their active period (dawn signals). Both photo-red and far-red lights failed to advance heart rate circadian phases at the late active period (e.g., CT 22) vs DD condition (Fig 2, S6 Fig). Animals continued their free-run circadian rhythms and delayed their circadian onset day by day, according to their individual intrinsic free-run circadian periods. However, later when the 12-h far-red fell into the range of early active period (e.g., CT 16), it induced circadian phase delays. It was assumed that 12-h photo-red would induce circadian phase delays as well if it was placed at the early active period. In addition, a prominent masking effect was observed in HR and locomotor activity rhythms in rats under 12-h photo-red LED light, while masking was less noticeable under 12-h far-red (Fig 2D and S4B Fig). In C57BL/6J mice, 12-h photo-red light, but not 12-h far-red light, induced body temperature circadian phase delay when the test lights were placed at the early active period (dusk signals) (Fig 2C, S4A Fig). Furthermore, it was shown that constant far-red LED lights increased free-run HR circadian period in Wistar rats, but not in C57BL/6J mice, when compared to DD condition (Fig 3C and 3D). These findings indicate that far-red light at 50 µW/cm^2^ would not entrain C57BL/6J mice and Wistar Han rats to a 12:12h light:dark cycle. Nevertheless, it may delay circadian phases and exhibit masking effect in rats; and Wistar Han rats are more sensitive to far-red LED light exposures than C57BL/6J mice.

Exposures of far-red LED during the dark phase induced alterations in circadian profiles in both mice and rats. Exposure of 2-h far-red at night induced transient alteration in activity, body temperature, and heart rate circadian profiles in mice and rats (Fig 4, S4 and S7 Figs) and exposure of 12-h far-red at night reduced HR circadian amplitude in rats (Fig 5J).

To better understand the mechanism involved in the red light induced changes in circadian rhythms, we recorded retinal dark-adapted and light-adapted ERG responses in C57BL/6J mice and Wistar Han rats. The 741 nm far-red LED light flashes (5 ms at 420–4200 µW/cm^2^) elicited significant dark-adapted ERG responses, but no light-adapted ERG responses, in both C57BL/6J mice and Wistar Han rats. The magnitude of dark-adapted ERG responses to the far-red LED flashes was much greater in Wistar Han rats than C57BL/6J mice. No ERG signal was detected from rodents when 850 nm infra-red LED light was used as stimulus flashes. The 666 nm photo-red LED flashes elicited both dark-adapted and light-adapted ERG responses in mice and rats, which is consistent with a previous study [22].

Rods and cones are involved in the retinal image-forming (IF) and non-image forming (NIF) functions [41,42]. While retinal ganglion cells (RGCs) convey rod-cone light input to the brain for visual image formation [41], the melanopsin-containing intrinsically photosensitive retinal ganglion cells (ipRGCs) relay rod-cone light input to suprachiasmatic nucleus (SCN) for non-image forming functions, which include light-induced circadian phase shift/photoentrainment/masking and pupillary reflex. IF vision was shown to be dissociated from NIF function at the retinal ganglion cell level, and mice with genetically ablated ipRGCs retain their IF vision, but lost NIF function [41]. In addition, IF vision in rodent is more sensitive to the dim-light and has lower threshold than NIF vision [42]. In our present study, 2-h far-red light at night induced a transient upstroke in locomotor activity and body temperature profiles in C57BL/6J mice, but no light-induced phase shift or masking was observed. In addition, when mice were exposed to a 3-h delayed light/dark cycle, far-red LED light did not cause any light-induced phase delays. Furthermore, constant far-red did not prolong free-run circadian period in mice vs DD. These data suggest that the 741 nm far-red light-induced transient changes in locomotor activity and body temperature profiles in mice are likely to be related to IF vision, not NIF function. This finding is in general consistent with a previous observation that rats reserve degraded object discrimination capacity under 729 nm LED illumination [35].

**Fig 8:**
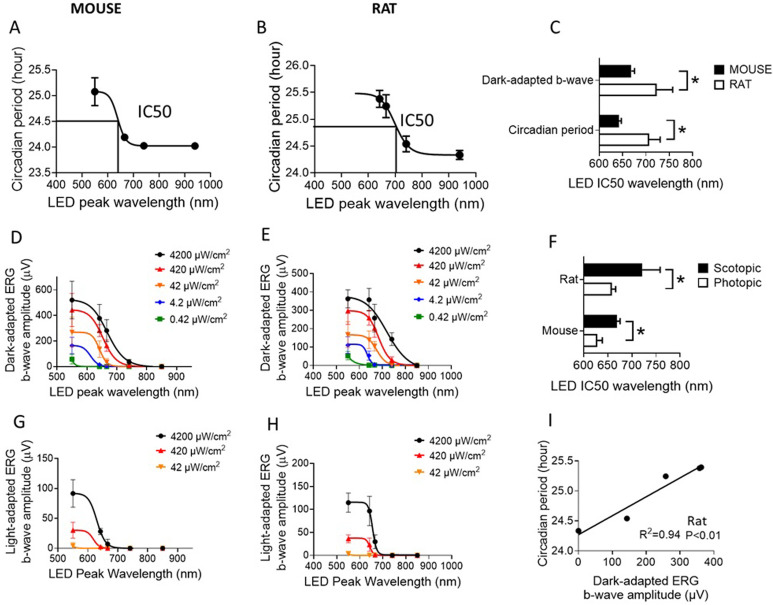
Free-run circadian period and ERG responses. **A)** Mouse body temperature free-run circadian period under different wavelength constant LEDs at 50 µW/cm^2^ (Aschoff effect). Assuming free-run circadian period under constant white light is equivalent to that under constant monochromatic 550 nm LED which is close to the maximum responses; and free-run circadian period under constant darkness is equivalent to that under constant 940 nm infra-red which is close to the minimum responses. LED IC50 wavelength was obtained through sigmoid fitting [Y = Bottom + (X^Hillslope)*(Top-Bottom)/(X^HillSlope + IC50^HillSlope)} the mean data. That is to say, LED light with a peak wavelength equal to IC50 will have 50% of maximum responses which correspond to white LED exposure. Greater IC50 wavelength means more sensitive to long wavelength LED exposures. **B)** Rat heart rate free-run circadian period under different wavelength constant LEDs at 50 µW/cm^2^ (Aschoff effect). **C)** LED IC50 wavelength obtained from free-run circadian period (under constant light at 50 µW/cm^2^) and dark-adapted b-wave amplitude (at 4200 µW/cm^2^ for 5 ms) – comparisons between mice and rats. **D)** LED wavelength dependent dark-adapted b-wave amplitude in C57BL/6J mice. Sigmoid fit was performed for each flash light intensity level. **E)** LED wavelength dependent dark-adapted b-wave amplitude in Wistar Han rats. **F)** LED IC50 wavelength from dark-adapted and light-adapted b-wave amplitudes (at 4200 µW/cm^2^ for 5 ms) – comparisons between dark-adapted and light-adapted responses. **G)** LED wavelength dependent light-adapted b-wave amplitude in C57BL/6J mice. **H)** LED wavelength dependent light-adapted b-wave amplitude in Wistar Han rats. **I)** Correlation between mean free-run circadian period (n = 8 at 50 µW/cm^2^ constant lights) and mean dark-adapted b-wave amplitude (n = 6 at 4200 µW/cm^2^ for 5 ms) obtained from Wistar Han rats with different wavelength LEDs. Each dot corresponds to each light source used. Data: mean ± SD. Circadian data N = 4 mice/8 rats; ERG data N = 7 mice/6 rats. *Significantly different between groups (P < 0.05, ANOVA).

What photoreceptors are involved in the circadian responses to red light in rodent? First, rods, cones, and ipRGCs are complementary in mediating NIF functions. Rods, cones, and ipRGCs photoreceptors together were shown to provide all of the photic input for NIF functions [43]. When both rod-cone and ipRGCs melanopsin phototransduction systems were silenced (triple-knock), mice failed to entrain to light/dark cycles, or to show any masking responses to light. Second, the melanopsin ipRGCs system alone is capable of providing attenuated NIF functions, such as circadian entrainment [44] and regulation of pineal melatonin [45] in mice lacking rods and cones (*rd/rd cl*). Third, the classical rod-cone system is also capable of providing attenuated NIF functions, such as light-induced circadian phase delays and lengthening of free-run circadian period in constant light in melanopsin knock-out (*Opn4*^*-/-*^) mice [46–48]. Forth, whilst rods and cones can mediate circadian responses in the absence of melanopsin, ipRGCs cells are required for both rod-cone and melanopsin pathways to provide NIF functions. Animals lacking ipRGCs cells retain pattern vision, but have deficits in circadian entrainment that resemble those observed in animals that lack phototransduction in all three classes [41]. The ipRGCs cells relay light and dark information from both rod-cone and melanopsin-based pathways to modulate sleep circadian rhythm [49]. Fifth, rods play a critical role in regulating circadian responses to red light. (i) Ablation of rods leads to loss of photosensitivity. Retinal degenerate CBA/J (*rd/rd*, loss of rods) mice were found to have substantially lower circadian photosensitivity (~ 100-fold less) for phase-shifting locomotor activity rhythms than normal CBA/N (+/+) mice [50,51]. (ii) Ablation of rods induces transition from primarily rods-mediated to primarily melanopsin-mediated circadian phase shift. The peak spectral sensitivity for circadian phase shifting was shifted from 500 nm with CBA/N (+/+) mice to 480 nm with CBA/J (rd/rd) mice, suggesting a transition from primarily rod-mediated (rhodopsin peak at 500 nm) to primarily melanopsin-mediated (melanopsin peak at 480 nm) response. (iii) Melanopsin photoreceptors have very limited contributions to NIF responses (e.g., circadian entrainment/phase shift) to red light in wild-type animals. Melanopsin has a peak absorption at 480 nm and insensitive to red light [9,52]. (iv) Cone photoreceptors may also have very limited contributions to NIF responses to red light in rodents. The substantial lower photosensitivity (100-fold less) in CBA/J (*rd/rd*) mice is due to loss of rod photoreceptors [51]. If cone photoreceptors were significantly involved, the remaining cones would sustain the photosensitivity in the retinal degenerate CBA/J (*rd/rd*) mice. (v) In fact, based on earlier observations it has long been hypothesized that retinal rod cells participate in the photic control of circadian rhythms that do not depend on perceived visual inputs [39,42,53]. (vi) In the present study, we found that far-red LED (peak wavelength 700–780 nm) will elicit dark-adapted rod (Fig 8D and 8E), but not light-adapted cone (Fig 8G and 8H), ERG responses in both mice and rats. In addition, it was noted that dark-adapted rod ERG responses were correlated with the circadian responses (e.g., lengthening of free-run circadian period in constant lights) in C57BL/6J mice and Wistar Han rats (Fig 8C and 8I). These data suggest the involvement of rod photoreceptors but not cone photoreceptors in far-red light induced circadian responses. Based on these evidences, it is tempting to hypothesize that the red light induced circadian responses are primarily mediated by rod photoreceptors and ipRGCs cells. Rod photoreceptors receive red light input and through synaptic network ipRGCs cells relay the information to SCN for circadian regulation.

The circadian photosensitivity to long wavelength red light in Wistar Han rats is significantly greater than C57BL/6J mice (Fig 8C). Both dark-adapted ERG and circadian responses were greater in Wistar Han rats than those in C57BL/6J mice. Exposure to 741 nm far-red induced circadian phase shift and lengthened free-run circadian period under constant light in the rats but not in the mice. Greater dark-adapted ERG responses to the far-red were observed in the rats than the mice, which is consistent with circadian responses. Female animals were not included in the present study due to a limited resource. The infra-red LEDs used in the circadian experiments (λ_p_ = 940 nm) are different from the ones used in the ERG experiments (λ_p_ = 850 nm) due to a technical reason.

**Fig 9:**
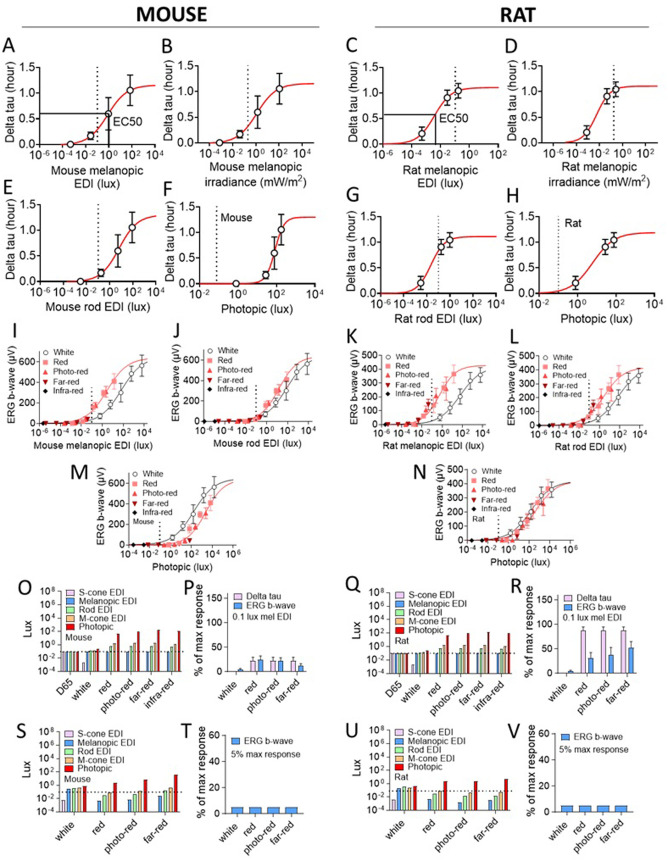
Quantification of species-specific α-opic light exposures associated with circadian and ERG responses in rodents. **A)** Mouse body temperature circadian responses plotted with mouse melanopic EDI (equivalent daylight illuminance). Circadian response (delta tau): lengthening of free-running circadian period under a constant test light (e.g., far-red, photo-red) vs constant dark (DD). Hill function y = b2+(b1-b2)/(1+(x/b3)^b4^) was used to fit the data obtained from the circadian experiment in individual animals to generate species-specific α-opic light exposures associated with 5% (EC5) and 50% (EC50) of maximum responses and circadian responses associated with species-specific 0.1 lux melanopic and 0.1 lux rod-opic EDI light exposures. **B)** Mouse delta tau plotted with mouse melanopic irradiance. **C)** Rat heart rate circadian responses (delta tau) plotted and fitted with rat melanopic EDI. **D)** Rat delta tau plotted with rat melanopic irradiance. **E)** Mouse delta tau plotted with mouse rod-opic EDI. **F)** Mouse delta tau plotted with human photopic illuminance (brightness to human eyes) of the light sources. **G)** Rat delta tau plotted with rat rod-opic EDI. **H)** Rat delta tau plotted with human photopic illuminance of the light sources. **I)**. Mouse dark-adapted ERG responses (b-wave amplitude) plotted with mouse melanopic EDI. **J)**. Mouse ERG b-wave amplitude plotted with mouse rod-opic EDI. **K)** Rat dark-adapted ERG b-wave amplitude plotted with rat melanopic EDI. **L)** Rat ERG b-wave amplitude plotted with rat rod-opic EDI. **M)** Mouse ERG b-wave amplitude plotted with human photopic illuminance of the light sources. **N)** Rat ERG b-wave amplitude plotted with human photopic illuminance of the light sources. **O)** Mouse α-opic EDIs at 0.1 lux melanopic EDI for D65 (standardized daylight), white, red, photo-red, far-red, and infra-red (850nm) LEDs. **P)** Mouse circadian (delta tau) and dark-adapted ERG (b-wave amplitude) responses at 0.1 lux melanopic EDI for different light sources. **Q)** Rat α-opic EDIs at 0.1 lux melanopic EDI for D65, white, red, photo-red, far-red, and infra-red (850nm) LEDs. **R)** Rat circadian (delta tau) and dark-adapted ERG (b-wave amplitude) responses at 0.1 lux melanopic EDI for different light sources. **S)** Mouse α-opic EDIs for white, red, photo-red, and far-red LEDs that associate with 5% of maximum dark-adapted ERG responses (ERG EC5). **T)**. Illustrate 5% of max ERG responses (ERG EC5) that correspond with panel S). **U)** Rat α-opic EDIs for white, red, photo-red, and far-red LEDs that associate with 5% of maximum dark-adapted ERG responses (ERG EC5). **V)**. Illustrate 5% of max ERG responses (ERG EC5) that correspond with panel **U)**. Dotted lines indicate recommended 0.1 lux melanopic EDI night light limit for lab animals. For D65, it would be 0.1 lux α-opic EDI for other opsins as shown in panel O) and Q). However, this is not the case for different long wavelength LEDs, which exhibit much greater rod and M-cone opic light exposures at 0.1 lux melanopic EDI. In addition, an α-opic measurement represents the level of light exposure to a particular opsin. However, this species-specific α-opic light exposure may not necessarily contribute to circadian or dark-adapted ERG responses. Data: mean ± SD. Circadian data: N = 4 mice/ 7 rats; ERG data: N = 6 mice/ 5 rats.

In addition, we took advantage of recent methods to calculate light intensity in quantities relevant for the species [54,55] to define a useful threshold for light to elicit a measurable response and to validate a recently proposed upper limit (i.e., 0.1 lux melanopic EDI) for rodent lighting at night [54]. Light intensities used in circadian and ERG experiments were quantified in species-specific α-opic irradiance, equivalent daylight illuminance (EDI), and human photopic illuminance using an online toolbox [55]. Circadian and ERG responses were plotted with associated species-specific α-opic measurements (Fig 9, S9–S12 Figs). Hill function y = b2+(b1-b2)/(1+(x/b3)b4) was used to fit the data obtained from individual animals to generate α-opic light exposures at EC5 and EC50 (i.e., 5% and 50% maximum responses, respectively) and the magnitude of responses at 0.1 lux melanopic and 0.1 lux rod-opic EDI light exposures (Fig 9, Tables 1 and 2).

**Table 1:** Quantification of species-specific α-opic light exposures associated with circadian responses in rodents.

Species	Mel EDI (lux)	Mel irradiance (mW/m^2^)	Rod EDI (lux)	Rod irradiance (mW/m^2^)	Human photopic (lux)	Delta tau (hour)	Delta tau (% of max)
Mouse	0.008 ± 0.006	0.012 ± 0.009	0.07 ± 0.05	0.11 ± 0.08	19 ± 6.3	0.06 ± 0.01	5
	1.1 ± 0.89	1.7 ± 1.4	7 ± 4.7	17 ± 15	94 ± 26	0.61 ± 0.13	50
	0.013 ± 0.004	0.017 ± 0.003	0.1	0.16	24 ± 1.7	0.09 ± 0.05	8.1 ± 4.7
	0.1	0.15	0.71 ± 0.13	1.4 ± 0.28	46 ± 2.6	0.26 ± 0.12	22 ± 7.2
Rat	0.0004 ± 0.0004^@^	0.0005 ± 0.0007^@^	0.002 ± 0.003^@^	0.002 ± 0.002^@^	0.33 ± 0.26^@^	0.06 ± 0.01	5
	0.01 ± 0.01^@^	0.01 ± 0.02^@^	0.058 ± 0.076^@^	0.075 ± 0.14^@^	5.6 ± 6.6^@^	0.58 ± 0.12	50
	1.3 ± 1.7	0.025 ± 0.003	0.1	0.16	39 ± 35	0.84 ± 0.18^@^	77 ± 26^@^
	0.1	0.15	1.5 ± 3.5	1.2 ± 0.42	28 ± 25	1.1 ± 0.08^@^	87 ± 7^@^

Species-specific α-opic light exposures associated with 5% and 50% of maximum responses (EC5 and EC50) and circadian responses at species-specific 0.1 lux melanopic and 0.1 lux rod-opic EDI light exposures. The endpoint used for circadian responses is the lengthening of free-running circadian period under a constant test light vs DD condition (i.e., delta tau). Free-running circadian period data were obtained from animals under DD, FF, PP, RR, and LL conditions. DD: constant dark. FF, PP, RR, and LL: constant far-red, photo-red, red, and white light, respectively. Mel: melanopic. EDI: α-opic equivalent daylight illuminance. α-Opic EDI describes the quantity of daylight (in human photopic illuminance lux) required to have that α-opic irradiance. Hill function y = b2+(b1-b2)/(1+(x/b3)^b4^) was used to fit the data obtained from the circadian experiment in individual animals to generate the measurements listed in the table. % of max: percent of maximum responses. An α-opic measurement represents the level of light exposure to a particular opsin. However, this species-specific α-opic light exposure may not necessarily contribute to circadian responses. Statistical analysis was performed for shaded data in the table between mice and rats.

@ Significantly different from respective measurements in mice (P < 0.05, ANOVA). Data: mean ± SD. N = 4 mice/ 7 rats.

**Table 2:** Quantification of species-specific α-opic light exposures associated with dark-adapted ERG responses in rodents.

Species	LEDs	Mel EDI (lux)	Mel irradiance (mW/m^2^)	Rod EDI (lux)	Rod irradiance (mW/m^2^)	Human photopic (lux)	ERG b-wave (µV)	ERG b-wave (% of max)
Mouse	White	0.30 ± 0.48	0.46 ± 0.74	0.38 ± 0.61	0.6 ± 1	0.76 ± 1.2	32 ± 7	5
		89 ± 78	136 ± 119	114 ± 99	179 ± 156	224 ± 195	317 ± 66	50
		0.078 ± 0.003	0.12	0.1	0.16	0.2	26 ± 13	4 ± 2
		0.1	0.15	0.13 ± 0.005	0.2	0.25	29 ± 15	4.5 ± 2.2
	Red	0.005 ± 0.004^*^	0.008 ± 0.005^*^	0.032 ± 0.023^*^	0.05 ± 0.04^*^	2.5 ± 1.8^*^	27 ± 7	5
		1.2 ± 1^*^	1.9 ± 1.5^*^	7.8 ± 6.4^*^	12 ± 10^*^	618 ± 505^*^	270 ± 63	50
		0.016	0.24	0.1	0.16	7.9	53 ± 8^*^	11 ± 3.6^*^
		0.1	0.15	0.63	1	50	123 ± 14^*^	24 ± 7.9^*^
	Photo-red	0.007 ± 0.007^*^	0.01 ± 0.01^*^	0.05 ± 0.05^*^	0.08 ± 0.07^*^	7.4 ± 7.1^*^	27 ± 6	5
		1.2 ± 0.8^*^	1.9 ± 1.3^*^	8.1 ± 5.5^*^	13 ± 8.7^*^	1268 ± 853^*^	267 ± 58	50
		0.015	0.023	0.1	0.16	16	46 ± 11^*^	9 ± 3^*^
		0.1	0.15	0.66	1	103	113 ± 23^*^	22 ± 6.6^*^
	Far-red	0.03 ± 0.01^*#$^	0.04 ± 0.02^*#$^	0.16 ± 0.07^#$^	0.25 ± 0.12^#$^	41 ± 18^*#$^	29 ± 9	5
		6.7 ± 10^*^	12 ± 15^*#$^	43 ± 66^*^	71 ± 105^*#^	4958 ± 4331^*#^	293 ± 87	50
		0.016	0.022 ± 0.002	0.1	0.16	27 ± 1.4	22 ± 6^#$^	4.0 ± 1.4^#$^
		0.1	0.015	0.64	0.91 ± 0.11	160 ± 17	79 ± 38^*#^	13 ± 3.4^*#$^
Rat	White	0.21 ± 0.23	0.31 ± 0.31	0.33 ± 0.26	0.38 ± 0.49	0.44 ± 0.55	20 ± 4	5
		64 ± 71	112 ± 136	97 ± 110	126 ± 145	205 ± 211	205 ± 37	50
		0.066 ± 0.034	0.23 ± 0.21	0.1	0.16	0.12 ± 0.1	13 ± 4.5	3.4 ± 1.6
		0.1	0.15	0.23 ± 0.24	0.16 ± 0.08	0.16 ± 0.1	18 ± 7.4	4.6 ± 2
	Red	0.005 ± 0.005^*^	0.008 ± 0.008^*^	0.029 ± 0.031^*^	0.047 ± 0.049^*^	2.4 ± 2.5^*^	20 ± 3.4	5
		0.52 ± 0.39^*^	0.76 ± 0.51^*^	3 ± 2.2^*^	4.6 ± 3.6^*^	212 ± 157	204 ± 36	50
		0.017	0.028 ± 0.003	0.1	0.16	8 ± 0.1	53 ± 21^*^	14 ± 5.9^*^
		0.1	0.15	0.59 ± 0.1	0.9 ± 0.16	44 ± 2.8	122 ± 39^*^	31 ± 11^*^
	Photo-red	0.002 ± 0.001^*@^	0.002 ± 0.001^*@^	0.016 ± 0.014^*^	0.009 ± 0.006^*#@^	0.84 ± 0.65^*@^	21 ± 3	5
		0.61 ± 0.51^*^	0.98 ± 0.8^*^	3.9 ± 3.5^*^	6.1 ± 5.6^*^	489 ± 465	205 ± 32	50
		0.013 ± 0.004	0.039 ± 0.02	0.1	0.16	8.5 ± 6.2	65 ± 31^*^	16 ± 7^*^
		0.1	0.15	0.83 ± 0.24	0.85 ± 0.32	75 ± 32	146 ± 59^*@^	38 ± 15^*@^
	Far-red	0.003 ± 0.003^*@^	0.003 ± 0.003^*@^	0.014 ± 0.01^*@^	0.029 ± 0.027^*@^	4.8 ± 3.9^*$@^	22 ± 4.2	5
		0.1 ± 0.07^*#$@^	0.24 ± 0.15^*@^	1 ± 0.89^*@^	1 ± 0.59^*#$@^	154 ± 92	221 ± 42	50
		0.017 ± 0.003	0.024 ± 0.004	0.1	0.16	25 ± 3.1	89 ± 30^*#@^	21 ± 7.9^*@^
		0.1	0.15	0.91 ± 0.36	0.76 ± 0.22	156 ± 16	210 ± 42^*#$@^	52 ± 13^*#$@^

Species-specific α-opic light exposures associated with 5% and 50% of maximum responses (EC5 and EC50) and dark-adapted ERG responses associated with species-specific 0.1 lux melanopic and 0.1 lux rod-opic EDI light exposures. Dark-adapted ERG responses: b-wave amplitude. Mel: melanopic. EDI: α-opic equivalent daylight illuminance. α-Opic EDI describes the quantity of daylight (in human photopic illuminance lux) required to produce that α-opic irradiance. Hill function y = b2+(b1-b2)/(1+(x/b3)^b4^) was used to fit the data obtained from the ERG experiment in individual animals to generate the measurements listed in the table. % of max: percent of maximum responses. An α-opic measurement represents the level of light exposure to a particular opsin. However, this species-specific α-opic light exposure may not necessarily contribute to dark-adapted ERG responses. Statistical analysis was performed for shaded data in the table.

*P < 0.05 vs respective white light;

#P < 0.05 vs respective red light;

$P < 0.05 vs respective photo-red light;

@P < 0.05 rat vs mouse.

ANOVA. Data: mean ± SD. N = 6 mice/ 5 rats.

*“Red” LEDs at 0.1 lux melanopic EDI elicit significant circadian responses in rodent:* The α-opic measurements associated with EC5 (i.e., 5% of maximum response) are considered as the thresholds for a specific light source to elicit measurable responses. From circadian study, we found that constant photo-red LED at 0.008 lux mouse melanopic EDI (or 0.012 mW/m^2^ mouse melanopic irradiance) and constant far-red LED at 0.0004 lux rat melanopic EDI (or 0.0005 mW/m^2^ rat melanopic irradiance) would lengthen free-run circadian period and produce a measurable circadian response in mouse and rat, respectively (Fig 9A–9D, Table 1). Whereas constant photo-red LED at 0.1 lux melanopic EDI (or 0.15 mW/m^2^ melanopic irradiance) would lengthen free-run circadian period and produce 22% and 87% of max responses in mouse and rat, respectively (Fig 9A–9D, Table 1). Even at 0.1 lux rod-opic EDI (or 0.16 mW/m^2^ rod-opic irradiance), constant photo-red would elicit 8% and 77% of max circadian responses in mouse and rat, respectively. Indeed, exposures of these “red” lights in the dark phase of light/dark cycles induced similar circadian responses measured in circadian onset or amplitude in mice and rats (Figs 3 and 5J, S8D and S8E Fig). Nevertheless, “red” LEDs at 0.1 lux human photopic illuminance would not elicit measurable circadian responses in rodent (Table 1). In conclusion, far-red or photo-red LEDs at 0.1 lux melanopic EDI (equivalent to 0.15 mW/m^2^ melanopic EDI or approximately 28–46 human photopic lux) or 0.1 lux rod-opic EDI (equivalent to 0.16 mW/m^2^ rod-opic irradiance or approximately 24–39 human photopic lux) will elicit significant circadian responses, so that 0.1 lux melanopic or rod-opic EDI may not be appropriate to serve as the upper limit for rodent lighting at night with “red” LEDs.

*Far-red LED at 5 photopic lux induces a measurable ERG response in rat:* Dark-adapted and light-adapted ERG responses were plotted with α-opic light exposures of white, red, photo-red, far-red, and infra-red LED flashes in mice and rats (Fig 9, S9–S12 Figs). A species-specific α-opic measurement represents the level of light exposure to a particular opsin. However, the α-opic light exposure to a specific opsin itself may not necessarily contribute to the ERG responses observed. The responses may be elicited through α-opic light exposures to other opsins. For dark-adapted ERG, low intensity stimulus flashes induce dim light sensitive rod responses and high intensity flashes induce combined rod and cone responses [56]. For light-adapted ERG, rods are saturated by bright light and become less sensitive to light so that light flashes evoke cone responses. With melanopic and rod-opic EDI (or irradiance) measurements, the dark-adapted ERG response curves for “red” LEDs were left-shifted, and the melanopic and rod-opic thresholds for “red” LEDs to elicit a measurable ERG response (EC5 or 5% of max) were significantly decreased compared with white LED in mouse and rat (P < 0.05, ANOVA) (Table 2). However, with human photopic illuminance measurement, the dark-adapted ERG response curves for “red” LEDs were right-shifted, and the photopic thresholds for “red” LEDs to elicit a measurable ERG response were significantly increased compared with white LED in mouse and rat (P < 0.05, ANOVA) (Table 2). In mouse, the photopic threshold for far-red LED was 41 photopic lux in dark-adapted ERG responses, which was significantly greater than those for red (2.5 photopic lux) and photo-red LEDs (7.4 photopic lux) (P < 0.05, ANOVA). Similarly, in rat the photopic threshold for far-red LED was 4.8 (approximately 5) photopic lux in dark-adapted ERG responses, which was significantly greater than the threshold for photo-red LEDs (0.84 photopic lux) (P < 0.05, ANOVA). Interestingly, photopic thresholds for far-red and photo-red in dark-adapted ERG responses in rat were significantly smaller than those in mouse (P < 0.05, ANOVA), but not for white and red LEDs. These data demonstrated that “red” lights, particularly far-red LED under 2 photopic lux (equivalent to 0.001 lux rat melanopic EDI, 0.008 lux rod-opic EDI, or 0.02 lux M-cone opic EDI for far-red), may provide a reasonable human photopic illumination for animal caretakers to work in the dark phase of a reversed light/dark cycles while impact on animals’ circadian rhythms and the capacity of night vision are minimized in rodent. In this report, we provide a spectrum of circadian responses (e.g., Aschoff effect – lengthening of free-run circadian period at constant light) and spectrum of dark-adapted and light-adapted ERG responses data in commonly used mice and rats to better understand and manage the influence of red light at night on animal wellbeing and the potential impact on study data. It is evident that mice and rats are capable of perceiving all the long wavelengths of red lights that human is able to see (380–780 nm) [57]. Natural environments are not completely dark at night and none of the data presented here actually represent evidence of a detrimental effect of dim red light at night. Given the substantial advantages in studying rodents in their active phase, utilizing a dim far-red LED light with its peak wavelength around 740–760 nm at less than 2 lux human photopic illuminance might be an effective alternative to maintain a good circadian rhythm in laboratory rodents (Table 3). Keep in mind, rodents are even more photosensitive to red light exposure at night with their image-forming vision function when behavioral studies are involved. Now that we are convinced that rodents, particularly Wistar Han rats, are capable of perceiving far-red LEDs with peak wavelengths of 740–760 nm, it would be interesting to see what’s the light thresholds for non-observable influence in image-forming functions using continuous home cage monitoring systems [58,59].

**Table 3:** Free-run circadian period and dark-adapted ERG b-wave amplitude in rats.

LED Lights	Peak wavelength ± half peak width (nm)	Illuminance at 50 µW/cm^2^ (lux)	Visibility	Dark-adapted b-wave amplitude (µV) (n = 7)	Projected dark-adapted b-wave amplitude (µV)	Lengthened free-run circadian period vs DD (hr)	Projected lengthened free-run circadian period vs DD (hr)
White		179	Very good visibility	363 ± 49 ^*^	364 ± 47 ^*^		1.13 ± 0.14 ^*^
642 nm Red	642 ± 10	87	Good visibility	358 ± 62 ^*^	318 ± 53 ^*^	1.04 ± 0.14 ^*^	1.05 ± 0.11 ^*^
666 nm photo-red	666 ± 11	26	Good visibility	258 ± 75 ^*^	264 ± 78 ^*^	0.91 ± 0.15 ^*^	0.90 ± 0.15 ^*^
741 nm far-red	741 ± 23	1	Limited visibility	143 ± 35 ^*^	119 ± 67 ^*^	0.21 ± 0.13 ^*^	0.18 ± 0.14 ^*^
750 nm far-red	750 ± 13	0.7	Poor visibility		96 ± 56 ^*^		0.13 ± 0.11 ^*^
760 nm far-red	760 ± 14	0.7	Poor visibility		75 ± 51 ^*^		0.09 ± 0.08 ^*^
770 nm far-red	770 ± 14	0.5	Very poor visibility		60 ± 46 ^*^		0.06 ± 0.07 ^*^
780 nm far-red	780 ± 15	< 0.5	Very poor visibility		49 ± 39 ^*^		0.04 ± 0.05 ^*^
850 nm infra-red	850 ± 9	0	Visible to LED bulb only	0	0		0.01 ± 0.01
940 nm infra-red	940 ± 26	0	Invisible			0	0

* Significantly different from infra-red (P < 0.05). Dark-adapted ERG was obtained at 4200 µW/cm^2^ (5 ms flash) (n = 6). Free-run circadian period was obtained at 50 µW/cm^2^ under constant light (n = 7). The projected data were obtained through sigmoid fitting [Y = Bottom + (X^Hillslope)*(Top-Bottom)/(X^HillSlope + EC50^HillSlope)} the data from individual animals. DD: constant darkness. The 940 nm infra-red is equivalent to darkness. Data: mean ± SD.

## Supporting information

S1 Fig Electronic circuit. Diagram of the electronic circuit used to generate 5 ms LED light flashes in ERG experiment.
(JPG)

S2 Fig Circadian robustness. A) Body temperature and locomotor activity circadian robustness in C57BL/6J mice. B) Heart rate, blood pressure and locomotor activity circadian robustness in Wistar Han rats. C) Effect of cage change event on circadian robustness in mice. D) Effect of cage change event on circadian robustness in rats. E) Mouse circadian robustness at different age. F) Rat circadian robustness at different age. Circadian robustness was calculated from 5-day 15-min mean dataset. ACT = locomotor activity; TEMP = body temperature; HR = heart rate; BP = blood pressure. Data: mean ± SD. N = 4 mice/ 7 rats. *Significantly different between groups (P < 0.05, ANOVA).
(JPG)

S3 Fig Circadian amplitude, acrophase, and mesor. Body temperature and locomotor activity A) circadian amplitude, C) circadian acrophase, and E) circadian mesor in C57BL/6J mice at different age. Heart rate, blood pressure and locomotor activity B) circadian amplitude, D) circadian acrophase, and F) circadian mesor in Wistar Han rats at different age. ACT = locomotor activity; TEMP = body temperature; HR = heart rate; BP = blood pressure. Data: mean ± SD. N = 4 mice/ 7 rats.
(JPG)

S4 Fig Locomotor activity actograms. A & C & E) Double-plotted mouse locomotor activity actograms. B & D & F) Double-plotted rat locomotor activity actograms. LD = 12:12h white light:dark; FD = 12:12h far-red light:dark; PD = 12:12h photo-red light:dark; DD = 24h constant darkness. LD + 2hF = 2h far-red in dark phase of a standard light/dark cycle; LF = 12:12h white light:far-red. Open rectangle box = test light-on period.
(JPG)

S5 Fig Mouse partial phase response curve obtained from entrainment protocol. A) Mouse body temperature double-plotted actogram showing onset phase shifts during the entrainment process by delayed 12:12h light/dark cycles. B) Diagram to illustrate onset phase shift during entrainment process. CT: circadian time. C) Daily onset phase shifts obtained from individual animals (offset with free-running rhythm in DD condition) by advanced and delayed 12:12h light/dark cycles. D) Daily onset phase shifts for individual animals were binned into 2-hour CT intervals. E) Actograms showing onset phase shifts by delayed 12:12h light/dark cycles (including 12:12h photo-red:dark cycles). F) Daily onset phase shifts obtained from individual animals by delayed 12:12h light/dark cycles with different LEDs. The phase response curve obtained from 12:12h light/dark entrainment protocol is different from those obtained from a standard phase response curve protocol where animals were exposed to a brief light pulse at different circadian time during constant dark and then measuring the phase shifts induced. Red boxes: 12h light-on period. N = 4 mice.
(JPG)

S6 Fig Rat partial phase response curve obtained from entrainment protocol. A) Individual rats heart rate double-plotted actograms showing effects by advanced 12:12h photo-red or far-red/dark cycles. B) Rat heart rate actogram showing circadian onset during advanced 12:12h test light/dark protocol. C) Diagram illustrating phase shifts during the entrainment experiment. D) Mean heart rate circadian onset phase shifts (offset with free-running rhythm in DD condition) plotted against the mean circadian time for individual animals. There was technical difficulty in obtaining accurate daily onset phase shifts due to decreased heart rate circadian rhythm, thus only mean data were obtained for individual animals. Red boxes: 12h light-on period. N = 7 rats.
(JPG)

S7 Fig Two-hour test light in the dark phase. A) Amplitude of mouse body temperature biphasic or monophasic waves induced by 2-hour test lights. B&C) Amplitude of rat HR biphasic/monophase waves induced by 2-hour test lights. D) Mouse body temperature difference in dark phase vs light phase (mean in dark phase – mean in light phase). E) Mouse body temperature circadian onset. F) Rat heart rate difference in dark phase vs light phase. *Significantly different from all other groups (P < 0.05, ANOVA). Data: mean ± SD. N = 4 mice/ 7 rats.
(JPG)

S8 Fig Twelve-hour test light in the whole dark phase. A) Mouse mean body temperature during mid-night hours (ZT15-21h) (*P < 0.05 vs LD and LP groups, ANOVA for all tests). B) Rat mean heart rate during mid-night hours (ZT15-21h) (*P < 0.05 vs LD, LR, and LI; #P < 0.05 vs LD, LF, LP, and LI. NS: LI vs LD). NS: no significant difference. C) Mouse body temperature difference in dark phase vs light phase (mean in dark phase – mean in light phase) (* P < 0.05 vs other three groups). D) Rat heart rate difference in dark phase vs light phase (*P < 0.05 vs LD, LF, and LI. NS: LI vs LD). E) Mouse body temperature circadian onset (*P < 0.05 LP vs LD and LF; #P < 0.05 LR vs LD, LF, and LP, ANOVA). Data: mean ± SD. N = 4 mice/ 7 rats.
(JPG)

S9 Fig Species-specific α-opic EDIs plotted with dark-adapted ERG b-wave amplitude in rodents. (JPG)

S10 Fig Species-specific α-opic irradiance plotted with dark-adapted ERG b-wave amplitude in rodents. (JPG)

S11 Fig Species-specific α-opic EDIs plotted with light-adapted ERG b-wave amplitude in rodents. (JPG)

S12 Fig Species-specific α-opic irradiance plotted with light-adapted ERG b-wave amplitude in rodents. (JPG)

S1 Table Circadian parameters from C57BL/6J mice. (DOCX)

S2 Table Circadian parameters from Wistar Han rats. (DOCX)

S3 Table Dark-adapted and light-adapted electroretinogram from C57BL/6J mice. (DOCX)

S4 Table Dark-adapted and light-adapted electroretinogram from Wistar rats. (DOCX)

S1 File Figures and tables raw data. (XLSX)
