# Supplementary figures and images for: Far-red LED light alters circadian rhythms and elicits dark-adapted ERG responses in rodents
Source: PLoS One. 2025 Jul 1;20(7):e0326710. doi: 10.1371/journal.pone.0326710 (PMC12212518; doi:10.1371/journal.pone.0326710)

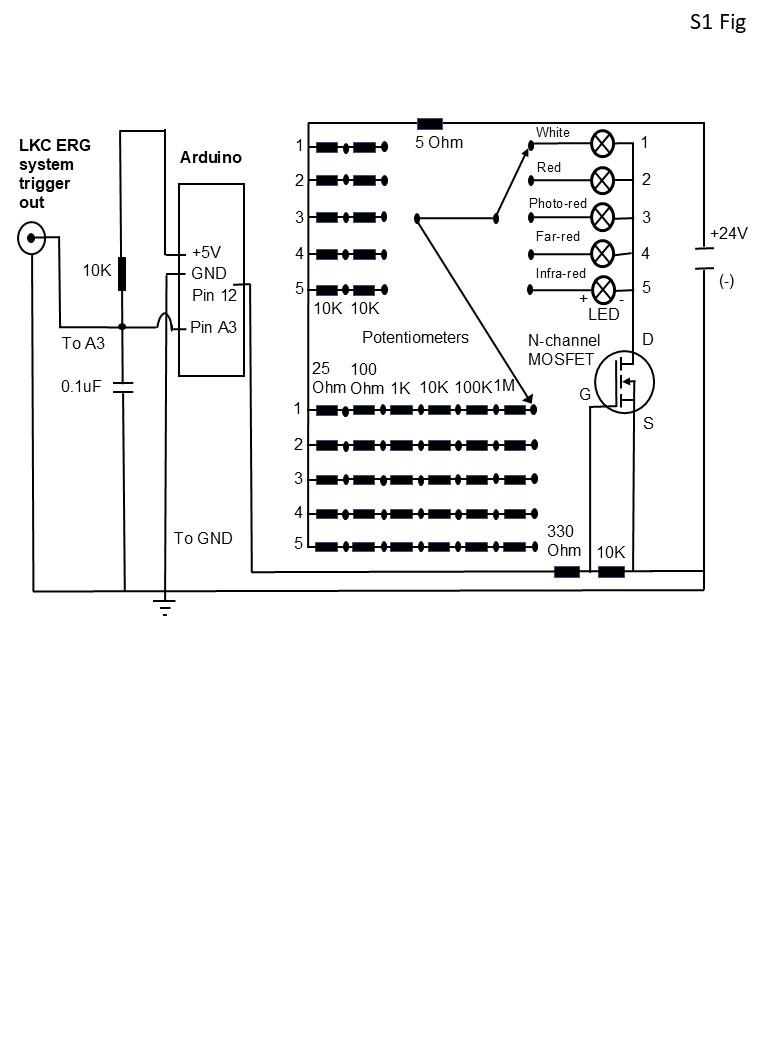

Supplement: S1 Fig — Diagram of the electronic circuit used to generate 5 ms LED light flashes in ERG experiment. (JPG) [file pone.0326710.s001.jpg]

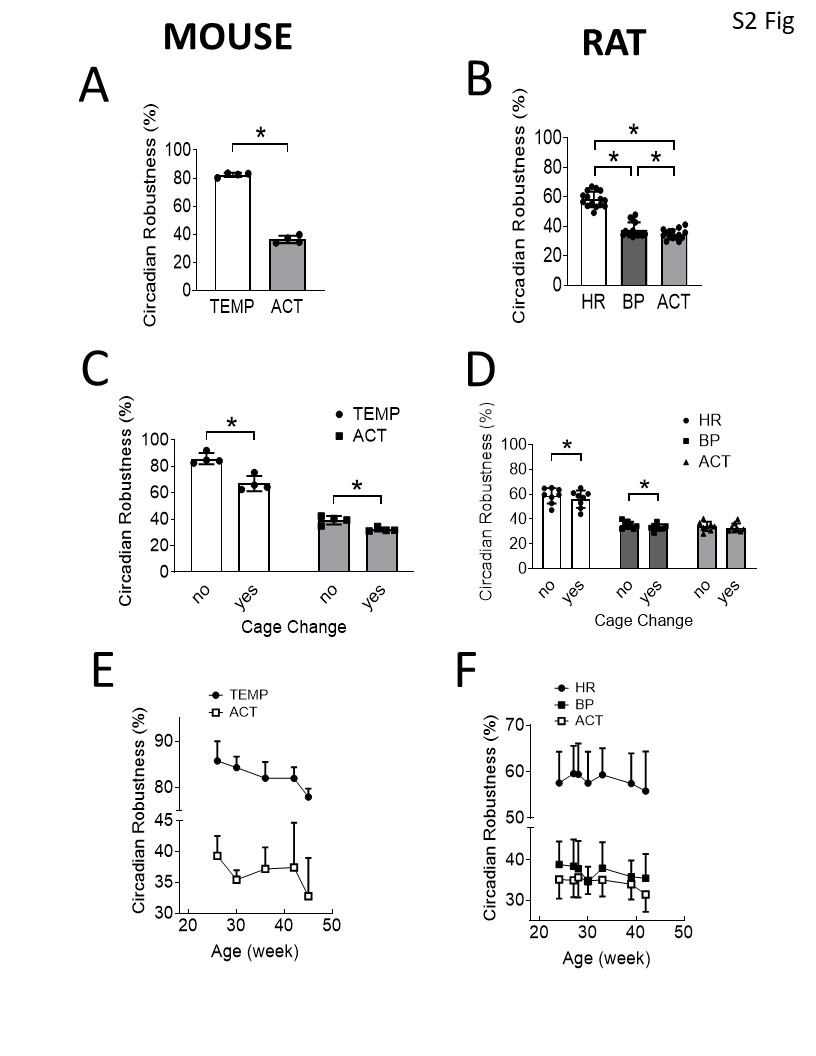

Supplement: S2 Fig — A) Body temperature and locomotor activity circadian robustness in C57BL/6J mice. B) Heart rate, blood pressure and locomotor activity circadian robustness in Wistar Han rats. C) Effect of cage change event on circadian robustness in mice. D) Effect of cage change event on circadian robustness in rats. E) Mouse circadian robustness at different age. F) Rat circadian robustness at different age. Circadian robustness was calculated from 5-day 15-min mean dataset. ACT = locomotor activity; TEMP = body temperature; HR = heart rate; BP = blood pressure. Data: mean ± SD. N = 4 mice/ 7 rats. *Significantly different between groups (P < 0.05, ANOVA). (JPG) [file pone.0326710.s002.jpg]

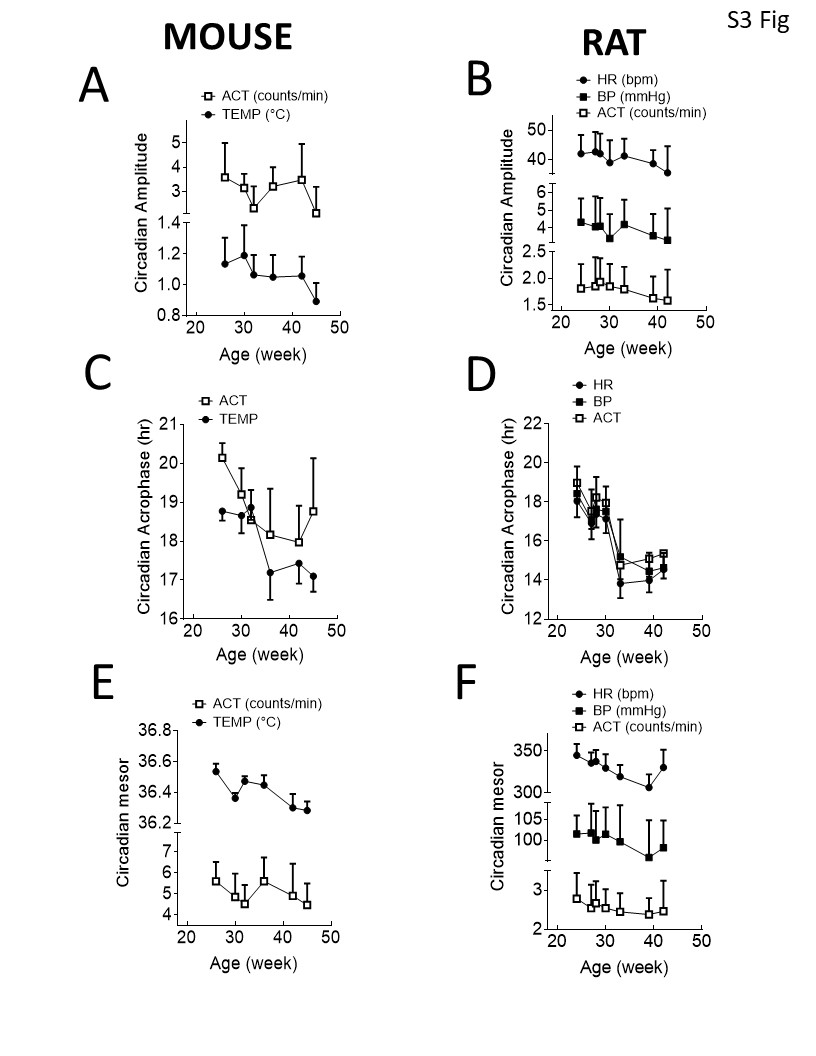

Supplement: S3 Fig — Body temperature and locomotor activity A) circadian amplitude, C) circadian acrophase, and E) circadian mesor in C57BL/6J mice at different age. Heart rate, blood pressure and locomotor activity B) circadian amplitude, D) circadian acrophase, and F) circadian mesor in Wistar Han rats at different age. ACT = locomotor activity; TEMP = body temperature; HR = heart rate; BP = blood pressure. Data: mean ± SD. N = 4 mice/ 7 rats. (JPG) [file pone.0326710.s003.jpg]

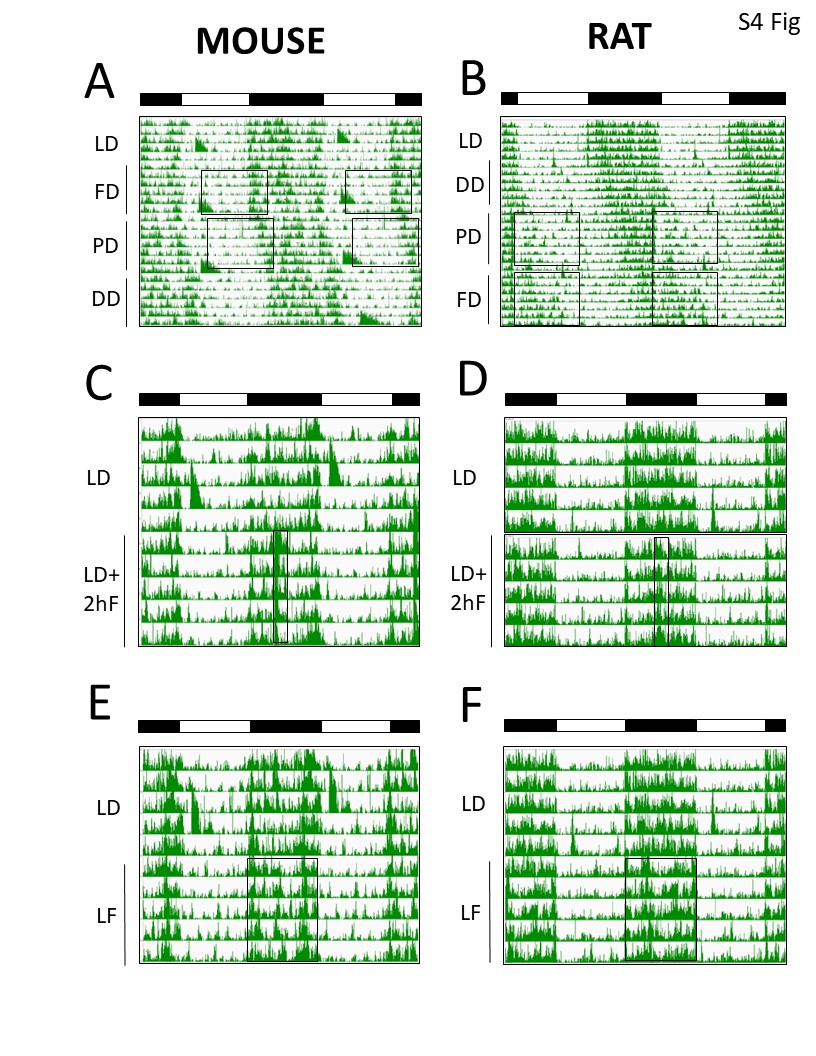

Supplement: S4 Fig — A & C & E) Double-plotted mouse locomotor activity actograms. B & D & F) Double-plotted rat locomotor activity actograms. LD = 12:12h white light:dark; FD = 12:12h far-red light:dark; PD = 12:12h photo-red light:dark; DD = 24h constant darkness. LD + 2hF = 2h far-red in dark phase of a standard light/dark cycle; LF = 12:12h white light:far-red. Open rectangle box = test light-on period. (JPG) [file pone.0326710.s004.jpg]

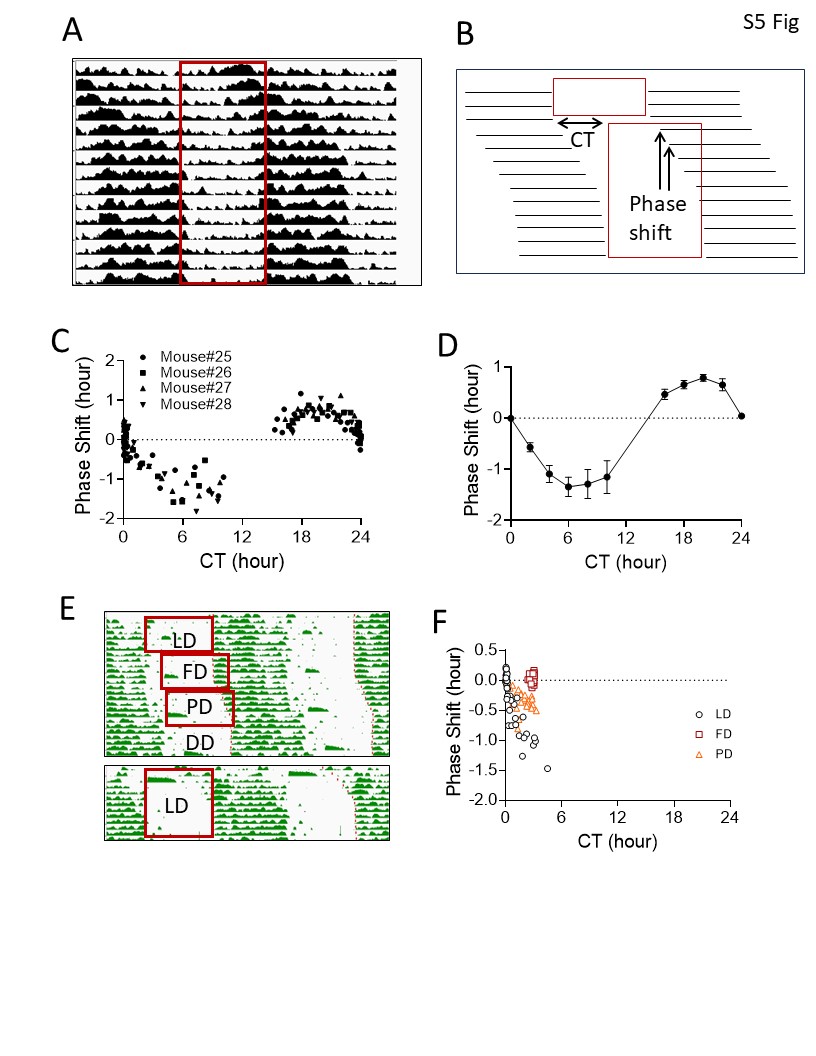

Supplement: S5 Fig — A) Mouse body temperature double-plotted actogram showing onset phase shifts during the entrainment process by delayed 12:12h light/dark cycles. B) Diagram to illustrate onset phase shift during entrainment process. CT: circadian time. C) Daily onset phase shifts obtained from individual animals (offset with free-running rhythm in DD condition) by advanced and delayed 12:12h light/dark cycles. D) Daily onset phase shifts for individual animals were binned into 2-hour CT intervals. E) Actograms showing onset phase shifts by delayed 12:12h light/dark cycles (including 12:12h photo-red:dark cycles). F) Daily onset phase shifts obtained from individual animals by delayed 12:12h light/dark cycles with different LEDs. The phase response curve obtained from 12:12h light/dark entrainment protocol is different from those obtained from a standard phase response curve protocol where animals were exposed to a brief light pulse at different circadian time during constant dark and then measuring the phase shifts induced. Red boxes: 12h light-on period. N = 4 mice. (JPG) [file pone.0326710.s005.jpg]

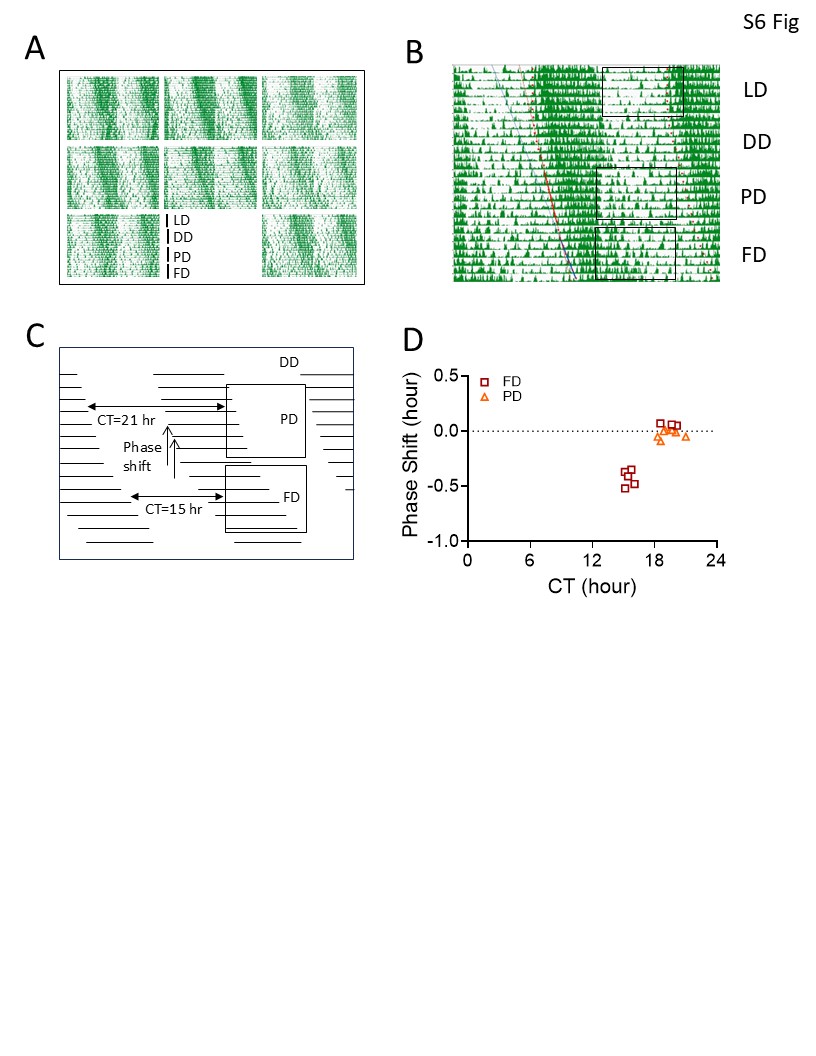

Supplement: S6 Fig — A) Individual rats heart rate double-plotted actograms showing effects by advanced 12:12h photo-red or far-red/dark cycles. B) Rat heart rate actogram showing circadian onset during advanced 12:12h test light/dark protocol. C) Diagram illustrating phase shifts during the entrainment experiment. D) Mean heart rate circadian onset phase shifts (offset with free-running rhythm in DD condition) plotted against the mean circadian time for individual animals. There was technical difficulty in obtaining accurate daily onset phase shifts due to decreased heart rate circadian rhythm, thus only mean data were obtained for individual animals. Red boxes: 12h light-on period. N = 7 rats. (JPG) [file pone.0326710.s006.jpg]

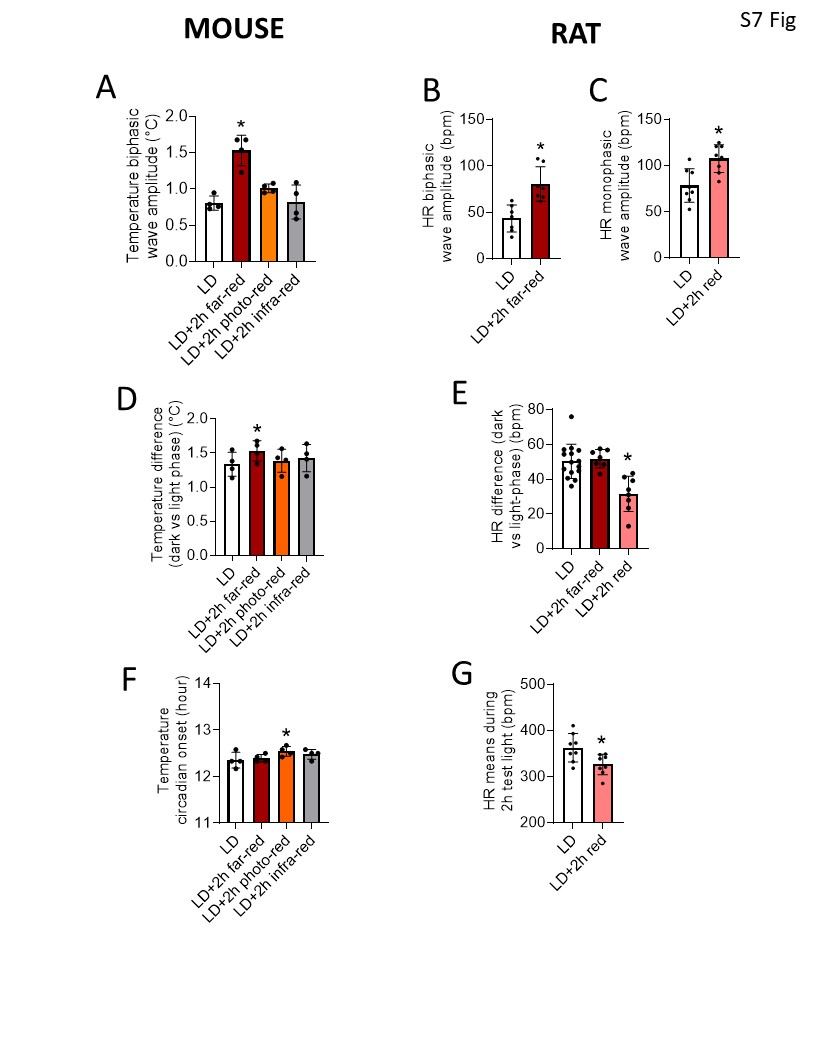

Supplement: S7 Fig — A) Amplitude of mouse body temperature biphasic or monophasic waves induced by 2-hour test lights. B&C) Amplitude of rat HR biphasic/monophase waves induced by 2-hour test lights. D) Mouse body temperature difference in dark phase vs light phase (mean in dark phase – mean in light phase). E) Mouse body temperature circadian onset. F) Rat heart rate difference in dark phase vs light phase. *Significantly different from all other groups (P < 0.05, ANOVA). Data: mean ± SD. N = 4 mice/ 7 rats. (JPG) [file pone.0326710.s007.jpg]

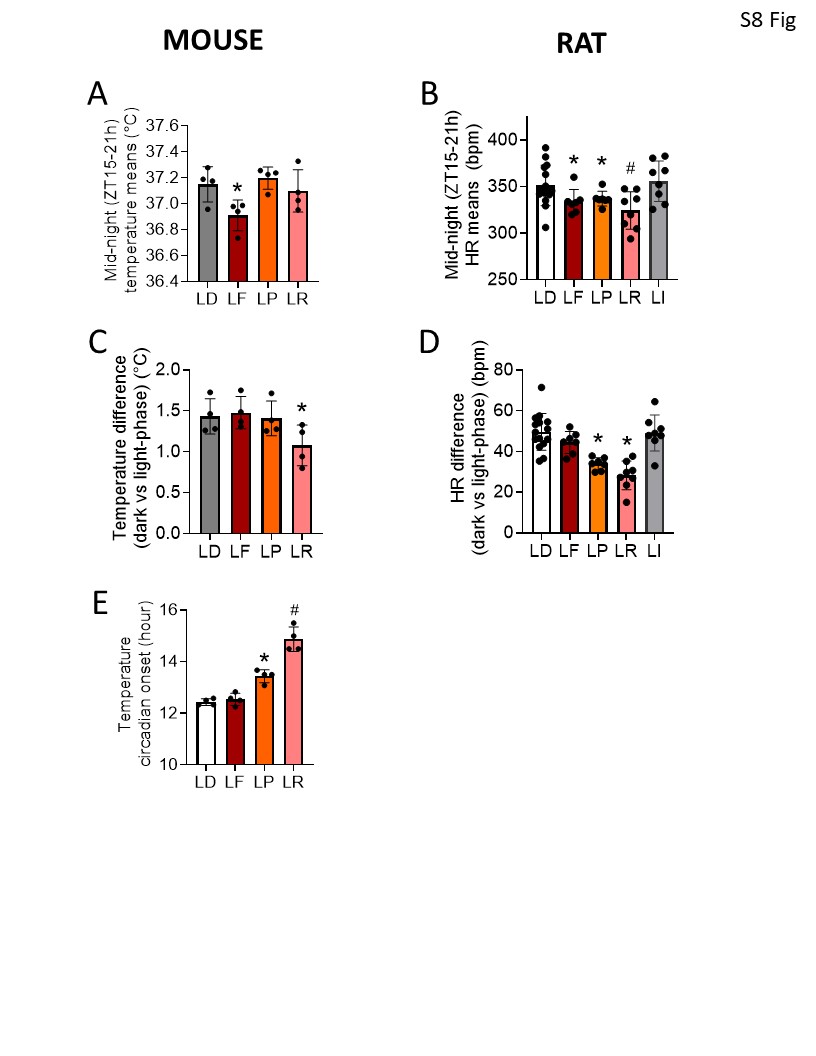

Supplement: S8 Fig — A) Mouse mean body temperature during mid-night hours (ZT15-21h) (*P < 0.05 vs LD and LP groups, ANOVA for all tests). B) Rat mean heart rate during mid-night hours (ZT15-21h) (*P < 0.05 vs LD, LR, and LI; #P < 0.05 vs LD, LF, LP, and LI. NS: LI vs LD). NS: no significant difference. C) Mouse body temperature difference in dark phase vs light phase (mean in dark phase – mean in light phase) (* P < 0.05 vs other three groups). D) Rat heart rate difference in dark phase vs light phase (*P < 0.05 vs LD, LF, and LI. NS: LI vs LD). E) Mouse body temperature circadian onset (*P < 0.05 LP vs LD and LF; #P < 0.05 LR vs LD, LF, and LP, ANOVA). Data: mean ± SD. N = 4 mice/ 7 rats. (JPG) [file pone.0326710.s008.jpg]

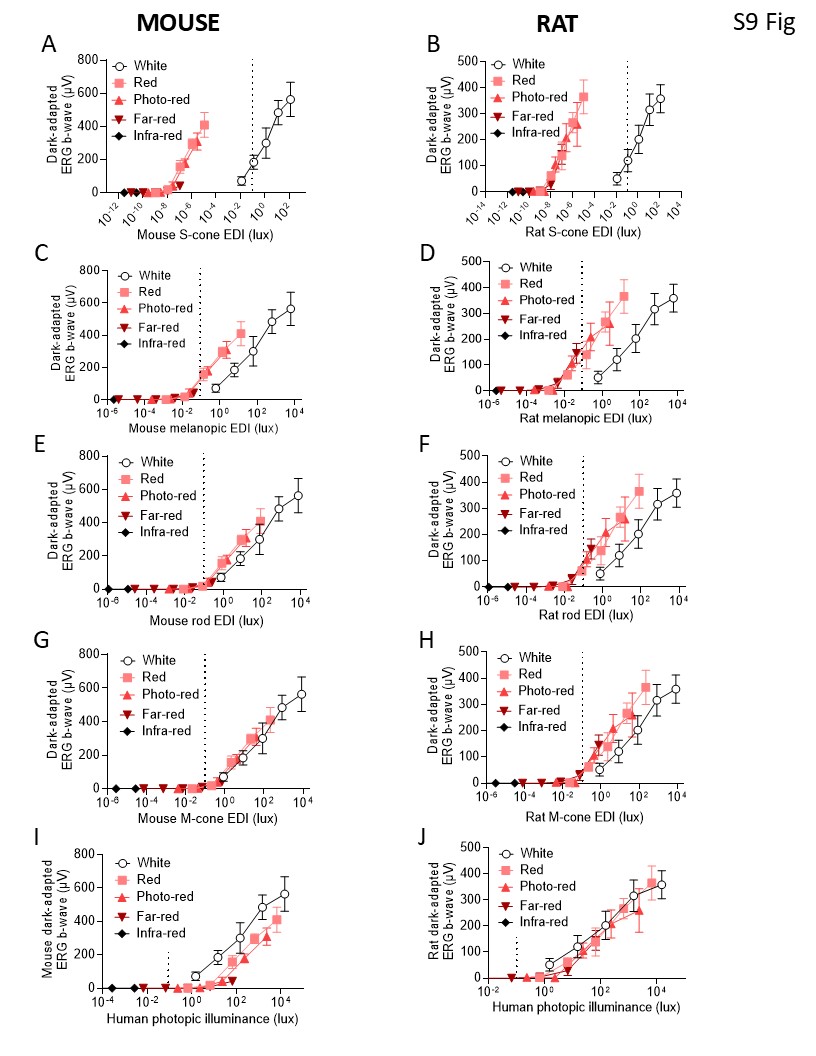

Supplement: S9 Fig — (JPG) [file pone.0326710.s009.jpg]

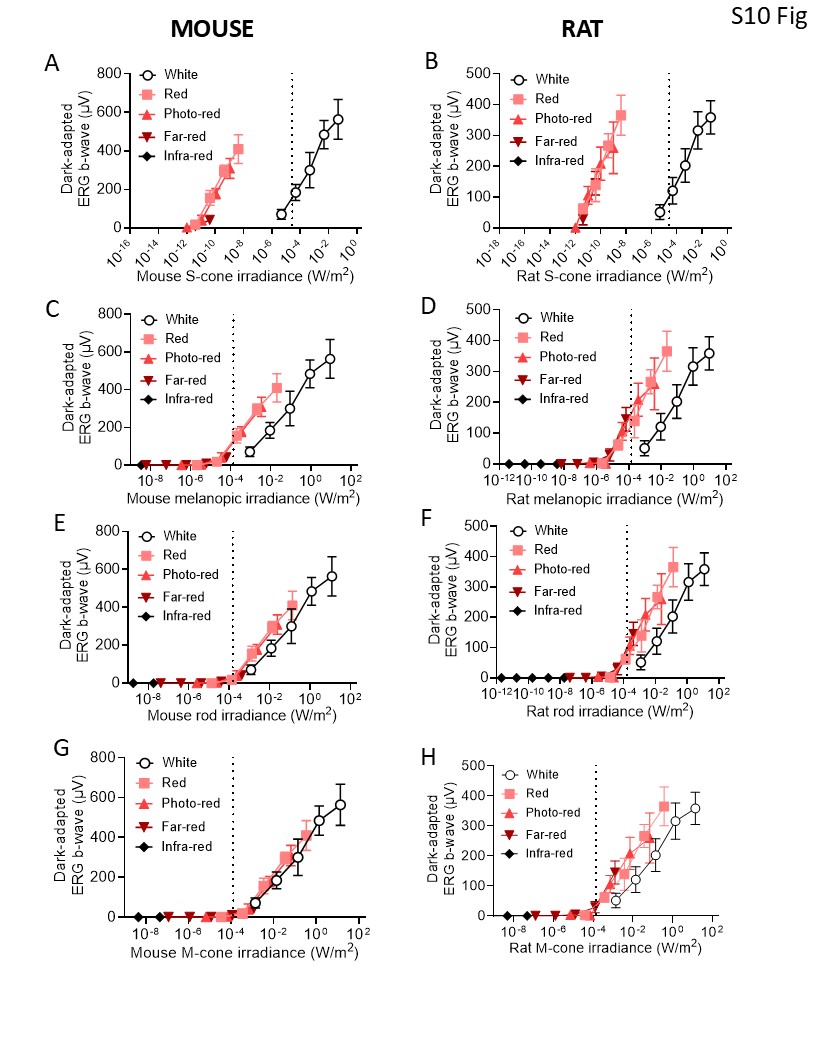

Supplement: S10 Fig — (JPG) [file pone.0326710.s010.jpg]

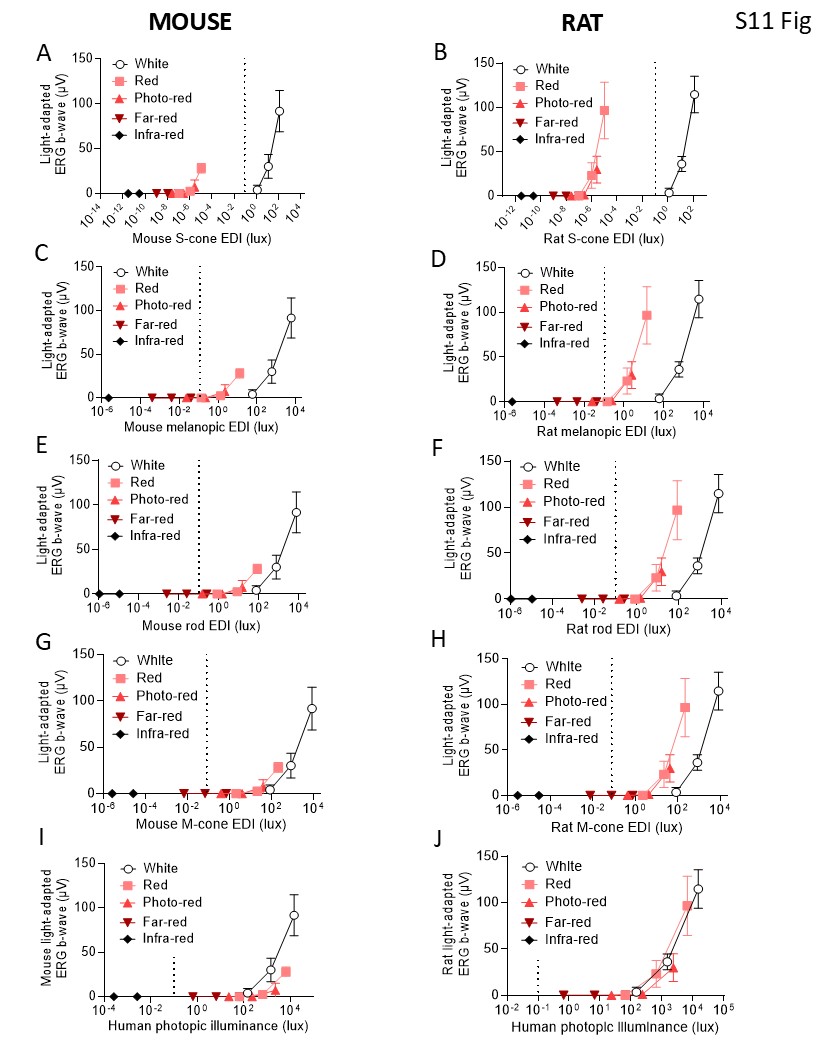

Supplement: S11 Fig — (JPG) [file pone.0326710.s011.jpg]

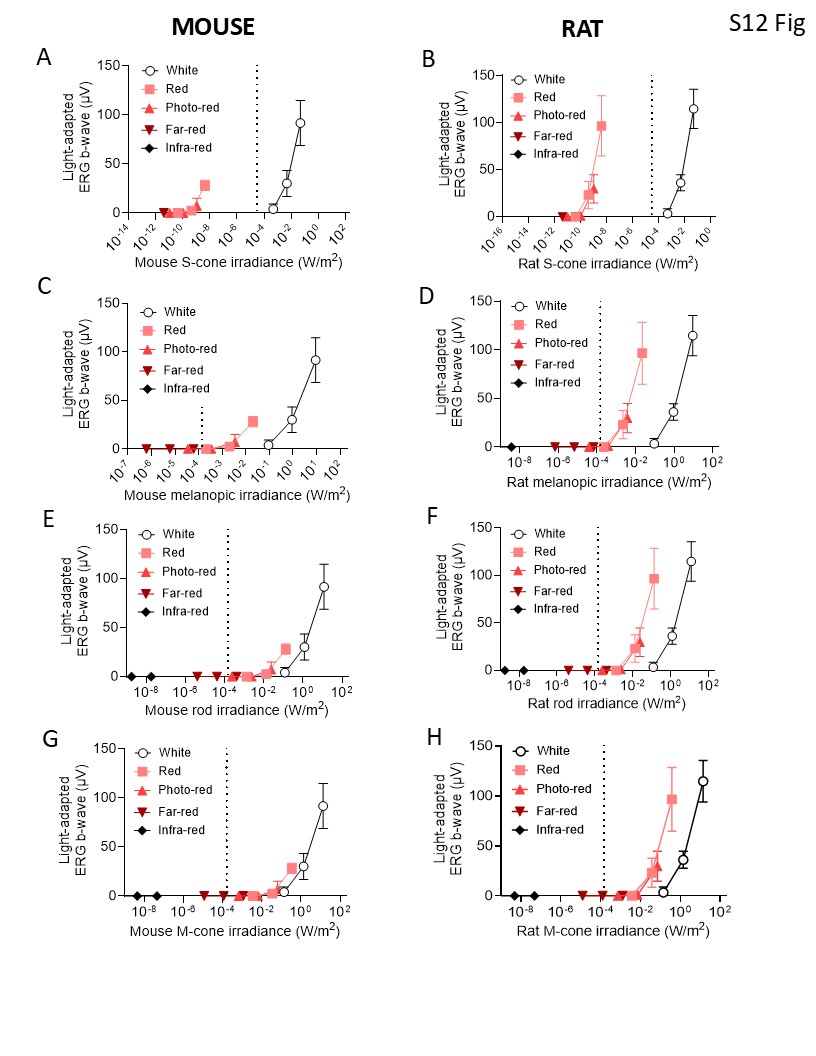

Supplement: S12 Fig — (JPG) [file pone.0326710.s012.jpg]
