# Supplementary material for: Far-red LED light alters circadian rhythms and elicits dark-adapted ERG responses in rodents
Source: PLoS One. 2025 Jul 1;20(7):e0326710. doi: 10.1371/journal.pone.0326710 (PMC12212518; doi:10.1371/journal.pone.0326710)
Supplement: S1 Table — (DOCX) [file pone.0326710.s013.docx]

**S1 Table. Circadian parameters from C57BL/6J mice**

|  | Circadian parameters | Light cycles | | | | | | | | | | | | |
| --- | --- | --- | --- | --- | --- | --- | --- | --- | --- | --- | --- | --- | --- | --- |
|  |  | LD | LD+2hI | LD+2hF | LD+2hP | LF | LP | LR | DD | FF | PP | LL | FD | PD |
|  |  | (n = 4) | (n = 4) | (n = 4) | (n = 4) | (n = 4) | (n = 4) | (n = 4) | (n = 4) | (n = 4) | (n = 4) | (n = 4) | (n = 4) | (n = 4) |
| Locomotor activity | Period (hr) | 24.29 | 24.29 | 24.13 | 24.46 | 24.63 | 24.51 | 24.36 | 24.57 | 24.11 | 24.84 | 25.31 | 24.65 | 25.06 |
|  |  | ±0.16 | ±0.25 | ±0.26 | ±0.27 | ±0.27 | ±0.27 | ±0.27 | ±0.38 | ±0.37 | ±0.36 | ±0.34*# | ±0.40 | ±0.39* |
|  | Amplitude (counts/min) | 2.42 | 3.09 | 3.73 | 2.84 | 2.43 | 2.52 | 2.79 | 2.36 | 1.92 | 2.31 | 2.26 | 1.75 | 1.94 |
|  |  | ±0.46 | ±0.58 | ±0.58* | ±0.60 | ±0.60 | ±0.60 | ±0.60 | ±0.78 | ±0.76 | ±0.75 | ±0.72 | ±0.81 | ±0.79 |
|  | Acrophase (hr) | 17.19 | 17.08 | 15.62 | 17.00 | 15.76 | 16.79 | 17.56 |  |  |  |  |  |  |
|  |  | ±0.45 | ±0.61 | ±0.63* | ±0.63 | ±0.66* | ±0.64 | ±0.61 |  |  |  |  |  |  |
|  | Mesor (counts/min) | 5.08 | 5.23 | 5.47 | 4.98 | 5.03 | 4.76 | 4.24 | 4.59 | 4.70 | 4.23 | 3.67 | 4.57 | 4.26 |
|  |  | ±0.48 | ±0.57 | ±0.57 | ±0.58 | ±0.59 | ±0.59 | ±0.58* | ±0.73 | ±0.71 | ±0.70 | ±0.68* | ±0.75 | ±0.74 |
|  | Robustness (%) | 33.14 | 31.25 | 37.80 | 32.23 | 33.44 | 30.73 | 32.14 | 29.55 | 31.53 | 34.39 | 32.81 | 29.25 | 28.41 |
|  |  | ±1.62 | ±2.40 | ±2.44 | ±2.53 | ±2.58 | ±2.54 | ±2.53 | ±3.57 | ±3.49 | ±3.41 | ±3.25 | ±3.74 | ±3.65 |
|  | Mean activity at light phase (counts/min) | 3.30 | 2.87 | 2.85 | 2.83 | 3.29 | 2.93 | 2.64 |  |  |  |  |  |  |
|  |  | ±0.36 | ±0.44 | ±0.45 | ±0.45 | ±0.47 | ±0.46 | ±0.44 |  |  |  |  |  |  |
|  | Mean activity at dark phase (counts/min) | 6.37 | 7.16 | 7.63 | 6.60 | 6.21 | 6.07 | 5.34 |  |  |  |  |  |  |
|  |  | ±0.89 | ±1.01 | ±1.03 | ±1.03 | ±1.05 | ±1.04 | ±1.01 |  |  |  |  |  |  |
| Body temperature | Period (hr) | 23.94 | 24.05 | 24.05 | 23.98 | 23.94 | 24.11 | 24.03 | 24.03 | 24.04 | 24.22 | 24.67 | 24.03 | 24.36 |
|  |  | ±0.05 | ±0.08 | ±0.08 | ±0.08 | ±0.09 | ±0.08* | ±0.08 | ±0.12 | ±0.12 | ±0.11*# | ±0.11*# | ±0.13 | ±0.12*# |
|  | Amplitude (°C) | 0.94 | 1.00 | 1.00 | 1.01 | 0.90 | 0.96 | 0.92 | 0.82 | 0.71 | 0.83 | 0.63 | 0.78 | 0.85 |
|  |  | ±0.07 | ±0.08 | ±0.08 | ±0.08 | ±0.08 | ±0.08 | ±0.08 | ±0.10 | ±0.10* | ±0.10 | ±0.10*# | ±0.11* | ±0.11 |
|  | Acrophase (hr) | 17.64 | 16.79 | 16.45 | 18.28 | 17.10 | 17.67 | 18.16 |  |  |  |  |  |  |
|  |  | ±0.23 | ±0.29* | ±0.29* | ±0.29* | ±0.30* | ±0.30 | ±0.29* |  |  |  |  |  |  |
|  | Mesor (°C) | 36.50 | 36.49 | 36.47 | 36.51 | 36.45 | 36.53 | 36.54 | 36.45 | 36.47 | 36.47 | 36.46 | 36.49 | 36.48 |
|  |  | ±0.03 | ±0.04 | ±0.04 | ±0.04 | ±0.04 | ±0.04 | ±0.04 | ±0.06 | ±0.06 | ±0.05 | ±0.05 | ±0.06 | ±0.06 |
|  | Robustness (%) | 73.03 | 74.01 | 73.90 | 72.92 | 68.92 | 71.76 | 66.14 | 65.39 | 64.03 | 66.66 | 55.28 | 64.90 | 65.43 |
|  |  | ±2.06 | ±2.83 | ±2.87 | ±2.96 | ±3.01 | ±2.97 | ±2.96* | ±4.04* | ±3.95* | ±3.87* | ±3.71*# | ±4.21* | ±4.12* |
|  | Mean temperature at light phase (°C) | 35.86 | 35.76 | 35.76 | 35.80 | 35.84 | 35.90 | 35.95 |  |  |  |  |  |  |
|  |  | ±0.06 | ±0.07 | ±0.07 | ±0.07 | ±0.07 | ±0.07 | ±0.07 |  |  |  |  |  |  |
|  | Mean temperature at dark phase (°C) | 37.04 | 37.10 | 37.07 | 37.12 | 36.95 | 37.05 | 37.01 |  |  |  |  |  |  |
|  |  | ±0.06 | ±0.07 | ±0.07 | ±0.07 | ±0.07 | ±0.07 | ±0.07 |  |  |  |  |  |  |
| *Significantly different from LD (P<0.05). #Significantly different from DD (P<0.05). Mixed Linear Model. Included all mouse circadian data. Data: Least squares means ± SEM. LD: 12:12h white light:dark cycle; LD+2hI, LD+2hF, and LD+2hP: 2h infra-red, far-red, and photo-red light in dark phase of LD cycle, respectively; LF, LP, and LR: 12:12h white light:far-red, white light:photo-red, and white light:red light cycle, respectively; DD, FF, PP, and LL: constant dark, far-red, photo-red, and white light, respectively. FD and PD: 3h delayed 12:12h far-red:dark and 3.7h delayed 12:12h photo-red:dark cycle, respectively. | | | | | | | | | | | | | | |
