# Supplementary material for: Far-red LED light alters circadian rhythms and elicits dark-adapted ERG responses in rodents
Source: PLoS One. 2025 Jul 1;20(7):e0326710. doi: 10.1371/journal.pone.0326710 (PMC12212518; doi:10.1371/journal.pone.0326710)
Supplement: S2 Table — (DOCX) [file pone.0326710.s014.docx]

**S2 Table. Circadian parameters from Wistar Han rats**

|  | Circadian parameters | Light cycles | | | | | | | | | | | | |
| --- | --- | --- | --- | --- | --- | --- | --- | --- | --- | --- | --- | --- | --- | --- |
|  |  | LD | LD +2hF | LD +2hR | LI | LF | LP | LR | DD | FF | PP | RR | FD | PD |
|  |  | (n = 15) | (n = 7) | (n = 8) | (n = 8) | (n = 7) | (n = 7) | (n = 8) | (n = 15) | (n = 7) | (n = 7) | (n = 7) | (n = 8) | (n = 8) |
| Locomotor activity | Period (hr) | 24.03 | 24.04 | 24.26 | 24.00 | 23.83 | 24.16 | 24.00 | 24.35 | 24.59 | 24.87 | 24.95 | 24.72 | 24.42 |
|  |  | ±0.08 | ±0.19 | ±0.18 | ±0.16 | ±0.17 | ±0.17 | ±0.16 | ±0.13* | ±0.17* | ±0.17*# | ±0.17*# | ±0.17*# | ±0.16* |
|  | Amplitude (counts/min) | 1.68 | 1.45 | 1.16 | 1.69 | 1.29 | 1.33 | 1.11 | 1.30 | 1.11 | 1.28 | 1.21 | 1.16 | 1.39 |
|  |  | ±0.09 | ±0.18 | ±0.16* | ±0.15 | ±0.15* | ±0.15* | ±0.15* | ±0.13* | ±0.16* | ±0.16* | ±0.15* | ±0.15* | ±0.15* |
|  | Acrophase (hr) | 16.74 | 17.94 | 16.02 | 16.40 | 16.20 | 17.68 | 18.30 |  |  |  |  |  |  |
|  |  | ±0.31 | ±0.71 | ±0.67 | ±0.60 | ±0.62 | ±0.62 | ±0.60* |  |  |  |  |  |  |
|  | Mesor (counts/min) | 2.50 | 2.34 | 2.11 | 2.45 | 2.41 | 2.24 | 2.18 | 2.42 | 2.44 | 2.35 | 2.25 | 2.24 | 2.39 |
|  |  | ±0.13 | ±0.21 | ±0.20* | ±0.19 | ±0.19 | ±0.19 | ±0.19 | ±0.16 | ±0.20 | ±0.20 | ±0.19 | ±0.19 | ±0.18 |
|  | Robustness (%) | 33.08 | 32.90 | 30.79 | 35.18 |  | 32.98 |  | 30.67 | 29.49 | 30.68 | 31.54 | 29.46 | 31.17 |
|  |  | ±0.79 | ±1.48 | ±1.36* | ±1.27 |  | ±1.27 |  | ±1.08* | ±1.38* | ±1.39* | ±1.28 | ±1.27* | ±1.26 |
|  | Mean activity at light phase (counts/min) | 1.57 | 1.33 | 1.56 | 1.49 | 1.66 | 1.35 | 1.55 |  |  |  |  |  |  |
|  |  | ±0.11 | ±0.21 | ±0.19 | ±0.18 | ±0.18 | ±0.18 | ±0.18 |  |  |  |  |  |  |
|  | Mean activity at dark phase (counts/min) | 3.42 | 3.32 | 2.65 | 3.42 | 3.18 | 3.11 | 2.82 |  |  |  |  |  |  |
|  |  | ±0.17 | ±0.33 | ±0.30* | ±0.28 | ±0.28 | ±0.28 | ±0.28 |  |  |  |  |  |  |
| Heart Rate | Period (hr) | 24.00 | 24.02 | 23.84 | 23.93 | 23.96 | 24.18 | 24.39 | 24.33 | 24.53 | 25.23 | 25.38 | 24.58 | 24.31 |
|  |  | ±0.03 | ±0.08 | ±0.08* | ±0.07 | ±0.07 | ±0.07* | ±0.07* | ±0.06* | ±0.08*# | ±0.08*# | ±0.07*# | ±0.07*# | ±0.07* |
|  | Amplitude (bpm) | 37.55 | 32.36 | 22.83 | 41.32 | 32.06 | 26.06 | 22.28 | 30.10 | 24.14 | 16.84 | 18.10 | 24.52 | 24.62 |
|  |  | ±1.42 | ±2.35* | ±2.16* | ±2.06 | ±2.05* | ±2.05* | ±2.06* | ±1.79* | ±2.21*# | ±2.22*# | ±2.07*# | ±2.05*# | ±2.03*# |
|  | Acrophase (hr) | 15.80 | 17.31 | 16.73 | 15.84 | 15.58 | 16.26 | 16.28 |  |  |  |  |  |  |
|  |  | ±0.23 | ±0.50* | ±0.45* | ±0.42 | ±0.42 | ±0.42 | ±0.42 |  |  |  |  |  |  |
|  | Mesor (bpm) | 332.39 | 341.70 | 344.27 | 329.23 | 321.20 | 319.89 | 325.48 | 332.30 | 332.93 | 330.89 | 308.27 | 327.69 | 324.80 |
|  |  | ±3.89 | ±5.24* | ±4.94* | ±4.79 | ±4.77* | ±4.77* | ±4.79 | ±4.39 | ±5.01 | ±5.04 | ±4.80*# | ±4.76 | ±4.74*# |
|  | Robustness (%) | 55.33 | 50.44 | 41.59 | 58.77 |  | 45.56 |  | 45.70 | 40.26 | 33.42 | 36.00 | 38.43 | 41.10 |
|  |  | ±1.46 | ±2.49* | ±2.29* | ±2.16 |  | ±2.16* |  | ±1.88* | ±2.33*# | ±2.35*# | ±2.17*# | ±2.15*# | ±2.14*# |
|  | Mean heart rate at light phase (bpm) | 314.15 | 320.46 | 329.13 | 306.31 | 304.53 | 303.08 | 310.29 |  |  |  |  |  |  |
|  |  | ±3.64 | ±5.17 | ±4.81 | ±4.67 | ±4.63* | ±4.63* | ±4.67 |  |  |  |  |  |  |
|  | Mean heart rate at dark phase (bpm) | 351.24 | 364.34 | 359.75 | 351.56 | 338.56 | 336.98 | 339.60 |  |  |  |  |  |  |
|  |  | ±4.49 | ±6.99* | ±6.43 | ±6.19 | ±6.13* | ±6.14* | ±6.19 |  |  |  |  |  |  |
| Blood pressure | Period (hr) | 24.01 | 24.25 | 24.53 | 23.96 | 23.98 | 24.40 | 24.27 | 24.60 | 24.08 | 24.79 | 25.00 | 24.36 | 25.00 |
|  |  | ±0.09 | ±0.19 | ±0.20* | ±0.17 | ±0.18 | ±0.17 | ±0.21 | ±0.14* | ±0.20# | ±0.18* | ±0.19* | ±0.17* | ±0.18*# |
|  | Amplitude (mmHg) | 3.70 | 2.76 | 2.56 | 3.74 | 2.41 | 1.76 | 2.84 | 2.78 | 2.04 | 2.39 | 2.56 | 2.61 | 2.08 |
|  |  | ±0.32 | ±0.47* | ±0.47* | ±0.43 | ±0.43* | ±0.42* | ±0.50 | ±0.38* | ±0.48* | ±0.45* | ±0.46* | ±0.42* | ±0.44* |
|  | Acrophase (hr) | 15.99 | 16.89 | 15.46 | 16.19 | 16.23 | 16.20 | 18.70 |  |  |  |  |  |  |
|  |  | ±0.37 | ±0.76 | ±0.76 | ±0.67 | ±0.68 | ±0.65 | ±0.83* |  |  |  |  |  |  |
|  | Mesor (mmHg) | 100.27 | 103.12 | 104.00 | 96.05 | 99.25 | 98.50 | 100.35 | 101.99 | 101.04 | 99.91 | 94.92 | 99.88 | 101.96 |
|  |  | ±2.06 | ±2.49* | ±2.47* | ±2.38* | ±2.38 | ±2.33 | ±2.58 | ±2.23* | ±2.53 | ±2.43 | ±2.46*# | ±2.33 | ±2.41 |
|  | Robustness (%) | 35.85 | 33.31 | 33.60 | 39.07 |  | 32.72 |  | 31.02 | 28.95 | 28.20 | 33.38 | 30.24 | 31.29 |
|  |  | ±1.19 | ±2.04 | ±2.02 | ±1.84 |  | ±1.76 |  | ±1.55* | ±2.10* | ±1.93* | ±1.98 | ±1.77* | ±1.90* |
|  | Mean blood pressure at light phase (mmHg) | 98.47 | 101.18 | 102.67 | 93.90 | 97.83 | 97.23 | 98.77 |  |  |  |  |  |  |
|  |  | ±1.95 | ±2.46 | ±2.43* | ±2.33* | ±2.32 | ±2.27 | ±2.56 |  |  |  |  |  |  |
|  | Mean blood pressure at dark phase (mmHg) | 102.16 | 104.95 | 105.65 | 98.40 | 100.77 | 99.66 | 102.46 |  |  |  |  |  |  |
|  |  | ±2.16 | ±2.73 | ±2.70* | ±2.58 | ±2.57 | ±2.51 | ±2.84 |  |  |  |  |  |  |
| *Significantly different from LD (P<0.05). #Significantly different from DD (P<0.05). Mixed Linear Model. Included all rat circadian data. Data: Least squares means ± SEM. LD: 12:12h white light-dark cycle; LD+2hF and LD+2hR: 2h far-red and red light in dark phase of LD cycle, respectively; LI, LF, LP, and LR: 12:12h white light:infra-red, white light:far-red, white light:photo-red, and white light:red light cycle, respectively; DD, FF, PP, and RR: constant dark, far-red, photo-red and red light, respectively. FD and PD: 5h advanced 12:12h far-red:dark and 3h advanced 12:12h photo-red:dark cycle, respectively. | | | | | | | | | | | | | | |
