# Supplementary material for: Far-red LED light alters circadian rhythms and elicits dark-adapted ERG responses in rodents
Source: PLoS One. 2025 Jul 1;20(7):e0326710. doi: 10.1371/journal.pone.0326710 (PMC12212518; doi:10.1371/journal.pone.0326710)
Supplement: S3 Table — (DOCX) [file pone.0326710.s015.docx]

**S3 Table. Dark-adapted and light-adapted electroretinogram from C57BL/6J mice**

| Parameter | Flash intensity (µW/cm^2^) | White light | Red light | Photo-red light | Far-red light | Infra-red light |
| --- | --- | --- | --- | --- | --- | --- |
| Dark-adapted b-wave amplitude (µV) | 0.42 | 57 ± 38 | 0 | 0 | 0 | 0 |
|  | 4.2 | 165 ± 65 | 15 ± 14 * | 2.7 ± 3.4 * | 0 * | 0 * |
|  | 42 | 269 ± 117 | 136 ± 64 * | 35 ± 28 *# | 0 *# | 0 *# |
|  | 420 | 443 ± 130 | 282 ± 47* | 164 ± 45 *# | 7.9 ± 7.1 *#$ | 0 *#$ |
|  | 4200 | 521 ± 147 | 377 ± 109 * | 282 ± 86 *# | 37 ± 19 *#$ | 0 *#$ |
| Dark-adapted b-wave latency (ms) | 0.42 | 76 ± 10 |  |  |  |  |
|  | 4.2 | 56 ± 5.2 | 87 ± 17 * | 84 ± 10 * |  |  |
|  | 42 | 38 ± 2.8 | 71 ± 5.7 * | 82 ± 7.0 *# |  |  |
|  | 420 | 33 ± 2.7 | 47 ± 3.0 * | 58 ± 6.9 *# | 78 ± 3.8 *#$ |  |
|  | 4200 | 30 ± 1.6 | 34 ± 1.5 | 45 ± 2.5 *# | 74 ± 4.2 *#$ |  |
| Light-adapted b-wave amplitude (µV) | 42 | 4.1 ± 5.3 | 0 | 0 | 0 | 0 |
|  | 420 | 30 ± 13 | 2.5 ± 3.7 * | 0 * | 0 * | 0 * |
|  | 4200 | 92 ± 23 | 28 ± 5.3 * | 7.2 ± 7.9 *# | 0 *# | 0 *# |
| Light-adapted b-wave latency (ms) | 42 | 41 ± 1.2 |  |  |  |  |
|  | 420 | 37 ± 3.1 | 48 ± 0 * |  |  |  |
|  | 4200 | 43 ± 1.3 | 35 ± 2.1 * | 44 ± 2.6 # |  |  |

*Significantly different from white light (P<0.05. #Significantly different from red light (P<0.05). $Significantly different from photo-red (P<0.05). @Significantly different from far-red (P<0.05). ANOVA. Included all mouse ERG data. Data: mean ± SD. N=7/group. Flash duration = 5 ms.
