# Supplementary material for: Far-red LED light alters circadian rhythms and elicits dark-adapted ERG responses in rodents
Source: PLoS One. 2025 Jul 1;20(7):e0326710. doi: 10.1371/journal.pone.0326710 (PMC12212518; doi:10.1371/journal.pone.0326710)
Supplement: S4 Table — (DOCX) [file pone.0326710.s016.docx]

**S4 Table. Dark-adapted and light-adapted electroretinogram from Wistar rats**

| Parameter | Flash intensity (µW/cm^2^) | White light | Red light | Photo-red light | Far-red light | Infra-red light |
| --- | --- | --- | --- | --- | --- | --- |
| Dark-adapted b-wave amplitude (µV) | 0.42 | 53 ± 22 | 1.6 ± 3.9 * | 2.6 ± 6.3 * | 0 * | 0 * |
|  | 4.2 | 111 ± 46 | 54 ± 25 * | 4.3 ± 7.7 *# | 0 *# | 0 *# |
|  | 42 | 168 ± 70 | 140 ± 48 | 105 ± 26 * | 4.1 ± 9.9 *#$ | 0 *#$ |
|  | 420 | 297 ± 73 | 259 ± 40 | 203 ± 50 *# | 30 ± 17 *#$ | 0 *#$ |
|  | 4200 | 363 ± 49 | 358 ± 62 | 258 ± 75 *# | 143 ± 35 *#$ | 0 *#$@ |
| Dark-adapted b-wave latency (ms) | 0.42 | 90 ± 4.57 |  |  |  |  |
|  | 4.2 | 67 ± 10 | 91 ± 8.4 * | 84.50 ± 6.36 * |  |  |
|  | 42 | 39 ± 1.5 | 60 ± 8.5 * | 84 ± 4.4 *# | 112 ± 0 *#$ |  |
|  | 420 | 42 ± 13 | 42 ± 7.2 | 44 ± 2.6 | 96 ± 5.2 *#$ |  |
|  | 4200 | 42 ± 15 | 41 ± 13 | 39 ± 6.1 | 80 ± 5.8 *#$ |  |
| Light-adapted b-wave amplitude (µV) | 42 | 3.5 ± 5.3 | 0 | 0 | 0 | 0 |
|  | 420 | 36 ± 8.6 | 23 ± 14 | 1.0 ± 2.5 *# | 0 *# | 0 *# |
|  | 4200 | 115 ± 21 | 97 ± 32 * | 30 ± 15 *# | 0 *#$ | 0 *#$ |
| Light-adapted b-wave latency (ms) | 42 | 39 ± 0.3 |  |  |  |  |
|  | 420 | 36 ± 2.3 | 37 ± 2.5 |  |  |  |
|  | 4200 | 45 ± 2.9 | 45 ± 1.7 | 37 ± 1.2 *# |  |  |

*Significantly different from white light (P<0.05). #Significantly different from red light (P<0.05). $Significantly different from photo-red (P<0.05). @Significantly different from far-red (P<0.05). ANOVA. Included all rat ERG data. Data: mean ± SD. N=6/group. Flash duration = 5 ms.
